# HCMV UL8 interaction with β-catenin and DVL2 regulates viral reactivation in CD34^+^ hematopoietic progenitor cells

**DOI:** 10.1128/jvi.01241-23

**Published:** 2023-09-29

**Authors:** Aaron Dirck, Nicole L. Diggins, Lindsey B. Crawford, Wilma D. Perez, Christopher J. Parkins, Hillary H. Struthers, Rebekah Turner, Andrew H. Pham, Jennifer Mitchell, Courtney R. Papen, Daniel Malouli, Meaghan H. Hancock, Patrizia Caposio

**Affiliations:** 1 Vaccine and Gene Therapy Institute, Oregon Health & Science University, Beaverton, Oregon, USA; The University of Arizona, Tucson, Arizona, USA

**Keywords:** human cytomegalovirus, UL8, viral reactivation, Wnt signaling

## Abstract

Human cytomegalovirus (HCMV) is a species-specific virus that establishes a persistent/latent infection in CD34^+^ hematopoietic progenitor cells (HPCs). The ability of HCMV to reactivate from latency is exquisitely linked to changes in cell signaling, which result in HPC differentiation. The Wnt/β-catenin pathway is tightly linked to CD34^+^ HPC homeostasis and differentiation. The majority of the viral and cellular factors involved in the maintenance of HCMV latency and reactivation are still unknown. Our group previously discovered a viral hematopoietic cytokine (pUL7) that promotes cellular differentiation and viral reactivation. Here, we show that the UL7-related RL11 family member UL8 is also required for efficient viral reactivation in CD34^+^ HPCs by interacting with components of the (Wnt)/β-catenin pathway. Pull-down experiments demonstrate that UL8 and β-catenin interact with Dishevelled-2 (DVL2) through their PDZ-binding domains, and this interaction promotes β-catenin stabilization and transcriptional activation. Disrupting the interaction among UL8, β-catenin, and DVL2 blocks HCMV reactivation *in vitro* and *in vivo*, similar to deletion of UL8. This is the first observation that Wnt signaling components are essential for HCMV reactivation, unlike α- and γ-herpesvirus that require an active Wnt pathway to maintain latency. Detailed understanding of how the Wnt network is manipulated by herpesviruses will improve our ability to develop targeted therapeutics.

**IMPORTANCE:**

CD34^+^ hematopoietic progenitor cells (HPCs) are an important cellular reservoir for latent human cytomegalovirus (HCMV). Several HCMV genes are expressed during latency that are involved with the maintenance of the viral genome in CD34^+^ HPC. However, little is known about the process of viral reactivation in these cells. Here, we describe a viral protein, pUL8, and its interaction and stabilization with members of the Wnt/β-catenin pathway as an important component of viral reactivation. We further define that pUL8 and β-catenin interact with DVL2 via a PDZ-binding domain, and loss of UL8 interaction with β-catenin-DVL2 restricts viral reactivation. Our findings will be instrumental in understanding the molecular processes involved in HCMV reactivation in order to design new antiviral therapeutics.

## INTRODUCTION

Human cytomegalovirus (HCMV) remains a significant cause of morbidity and mortality after solid organ transplant and hematopoietic stem cell transplant (1, 2) due to the ability of the virus to establish a latent infection in CD34^+^ hematopoietic progenitor cells (HPCs) (3 – 5). Latent infection is defined as the maintenance of the viral genome in the absence of production of infectious virions. New virions are produced upon reactivation, which correlates with a change in the differentiation state of the infected cells and is observed upon *ex vivo* terminal differentiation of either naturally or experimentally infected CD34^+^ HPCs into macrophages or dendritic cells (6). The mechanisms that regulate establishment and maintenance of HCMV latency as well as reactivation are poorly understood at the molecular level.

Wnt signaling and β-catenin activity, initially discovered by genetic analysis in the wing development of *Drosophila*, are necessary for proper function and development of hematopoietic cells (7, 8). Indeed, Wnt ligands and receptors activate a complex set of signaling pathways that control stem cell proliferation and differentiation (9). In the off state, β-catenin, the major effector protein in the canonical Wnt pathway, is localized in the cytoplasm bound to a multiprotein proteasomal destruction complex that promotes β-catenin phosphorylation and degradation (10). Canonical Wnt signaling is activated via Wnt ligands binding to their respective dimeric cell surface receptors composed of seven Frizzled proteins and the low-density lipoprotein receptor-related protein (LPR5/6) co-receptors. Upon Wnt stimulation, the cytoplasmic protein Dishevelled (Dvl) is recruited, phosphorylated, and activated. Activation of Dvl induces inhibition of the destruction complex and release of β-catenin. Subsequently, stabilized β-catenin translocates into the nucleus and acts as a coactivator of T cell specific factor (TCF)/lymphoid enhancer factor-1 transcription factor that regulates expression of genes involved in cell cycle regulation, cell migration, cell fate/differentiation, and stem cell maturation (9). α-Herpesvirus and γ-herpesvirus proteins are known to interact with components of the Wnt pathway to control latency during infection (11 – 16). In mice, cytomegalovirus (CMV)-induced Wnt signaling leads to increased mobility and invasiveness of bone marrow-derived macrophages (17). To date, the role of Wnt/β-catenin signaling in HCMV-infected CD34^+^ HPCs is unknown.

Our group has previously characterized HCMV pUL7 as a viral secreted factor essential for viral reactivation from latency *in vitro* and *in vivo* (18). We observed that pUL7 binds the Fms-like tyrosine kinase 3 receptor (Flt-3R), inducing signaling necessary to promote CD34^+^ HPC differentiation into monocytes during hematopoiesis (18). UL7 is partially colinear with another member of the RL11 gene family, UL8 (19). While pUL7 and pUL8 share an N-terminal Ig-like domain, pUL8 is a transmembrane protein with a longer N-terminal stalk domain and a distinctive cytoplasmic tail with two tyrosine-based endocytic motifs and a PDZ-binding domain at the carboxy terminus (19). In contrast to UL7, which is predominantly shed when ectopically expressed, UL8 is expressed on the cell surface and most likely functions as a receptor (19).

In the current study, we observed that deletion of UL8 impairs reactivation *in vitro* and *in vivo* because of the necessity of UL8 to interact with and stabilize components of the Wnt/β-catenin pathway. Indeed, treatment of HCMV latently infected CD34^+^ HPCs with the Wnt antagonist Dickkopf-1 (DKK-1) impairs viral reactivation. We also determined that the formation of the UL8-β-catenin-DVL2 complex promotes β-catenin stabilization and transcriptional activation. These results indicate that UL8 contribution to β-catenin stabilization and transcriptional activation is essential to activate the Wnt/β-catenin pathway to promote viral reactivation.

## RESULTS

### HCMV UL8 is required for HCMV reactivation

**Fig 1:**
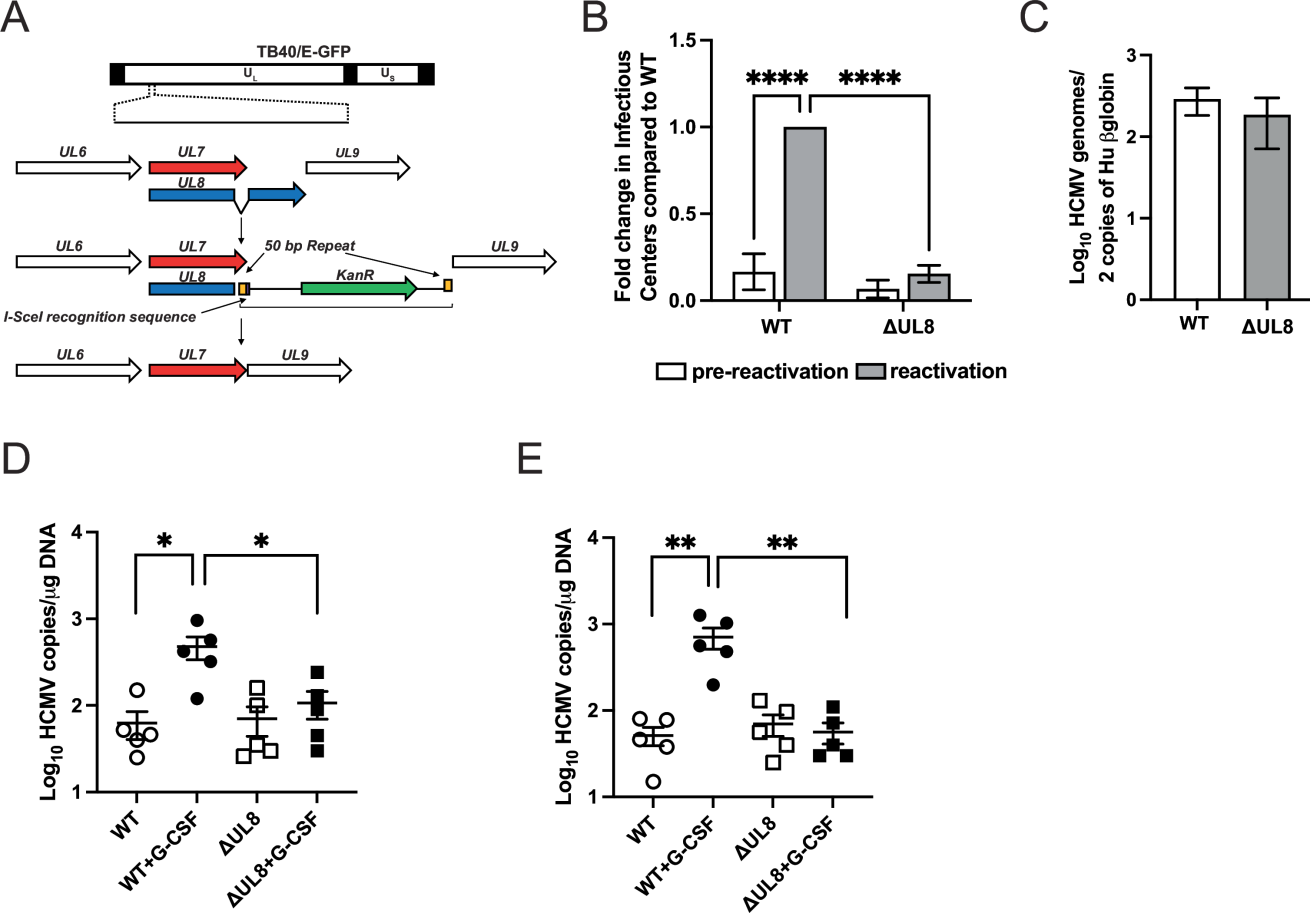
Deletion of UL8 prevents viral reactivation *in vitro* and *in vivo*. (**A**) Generation of HCMV TB40/EΔUL8 by en passant recombination. Briefly, we amplified a recombination insert consisting of an *I-SceI* recognition sequence (purple square) followed by an aminoglycoside 3′-phosphotransferase (kanamycin resistance, KanR) selection marker with a sense primer containing a 100-bp homology to the region just upstream of the UL7 termination codon and an antisense primer containing a 50-bp homology to the genomic region just downstream of the UL8 termination codon followed by 50 bp homologous to the region just upstream of the UL7 termination codon. Hence, 50 bp upstream of the UL7 termination codon was repeated in both primers (yellow squares), creating a 50-bp long repeated region flanking the recombination insert. The PCR product generated to replace exon 2 of ORF UL8 was inserted into the TB40 BAC backbone by homologous recombination through heat shock induction of the λ phage derived Red recombination genes in *E.coli* strain GS1783. (**B**) Pure populations of CD34^+^ HPCs infected with HCMV WT or ΔUL8 mutant were isolated by FACS at 48 hours post-infection and maintained in LTBMC medium. At 14 dpi, viable CD34^+^ HPCs were seeded onto fibroblast monolayers plated in 96-well dishes by extreme limiting dilution in cytokine-rich media for reactivation. An equivalent number of cells were mechanically disrupted and seeded in parallel to determine the infectious virus present in the cultures prior to reactivation (pre-reactivation). The frequency of infectious center formation pre- and post-reactivation was determined 14 days later from the number of GFP^+^ wells at each dilution using extreme limiting dilution analysis. Data shown in the fold change in reactivation normalized to HCMV WT post-reactivation. Bars represent the mean ± SEM of three independent experiments. Statistical significance was determined using two-way analysis of variance (ANOVA) with Tukey’s multiple comparison test (****P < 0.0001). (**C**) Total DNA from primary CD34^+^ HPC cultures was extracted at 14 dpi (12 days post-latency culture initiation, latency established), and HCMV genomes were quantified using quantitative PCR (qPCR) with primers and probe specific for the *UL141* gene. Viral genomes synthesized during infection in CD34^+^ HPCs were normalized to total cell number determined using human-globin as a reference. Data shown are the mean value for three replicate qPCRs, and error bars represent standard deviation. Data shown are representative of three independent experiments. (**D, E**) Sublethally irradiated NOD-*scid* IL2Rγ_c_^null^ mice were engrafted with CD34^+^ HPCs (huNSG) and subsequently injected with human fibroblasts previously infected with HCMV WT or ΔUL8 mutant as indicated. At 4 weeks post-infection, viral reactivation was triggered by treating latently infected HCMV WT and HCMVΔUL8 (*n* = 5) animals with G-CSF and AMD-3100. At 1 week post-treatment, mice were euthanized, and tissues were harvested. Total genomic DNA was isolated from spleen tissue (**D**) or liver tissues (**E**), and HCMV genomes were quantified using qPCR with primers and probe specific for the *UL141* gene. Statistical significance was determined using two-way ANOVA, followed by Bonferroni’s multiple comparison test (*P < 0.01*;* **P < 0.001).

We have previously shown that pUL7 is required for viral reactivation (18). Since pUL7 and pUL8 share the N-terminal domain Ig-like domain, we investigated whether UL8 was also required for reactivation. We constructed a UL8 deletion in HCMV TB40/E-GFP leaving UL7 intact (HCMVΔUL8) (Fig. 1A). Since pUL7 is secreted from infected cells (19), we evaluated the integrity of UL7 transcript by RT-PCR. As shown in S1A, using specific probes for UL7 and UL8, UL7 transcript is expressed both in HCMV WT- and HCMVΔUL8-infected cells, while UL8 transcript is only expressed in HCMV WT. Moreover, integrity of the HCMVΔUL8 BAC was assessed by whole genome next-generation sequencing (NSG) (S1B). Subsequently, human embrionic stem cell (hESC)-derived CD34^+^ HPCs were infected with HCMV WT or ΔUL8 for 48 hours, and viable CD34^+^ GFP^+^ cells were sorted by fluorescence-activated cell sorting (FACS), which were then seeded into long-term bone marrow culture (LTBMC) over stromal cell support (18). After 12 days of culture in LTBMC, HCMV latently infected HPCs were seeded onto monolayers of permissive fibroblasts in cytokine-rich media in an extreme limiting dilution assay to promote myeloid differentiation; an equivalent number of cells were lysed and plated similarly on fibroblasts to serve as a pre-reactivation control. As shown in Fig. 1B, we observed a significant reduction in infectious centers in ΔUL8-infected cells compared to WT (*P* < 0.0001). Analysis of viral DNA in both HCMV WT- and HCMVΔUL8-infected CD34^+^ HPCs at 14 days post-infection (dpi) indicated similar amounts of HCMV genomes in both cell populations (Fig. 1C; Fig. S2), demonstrating that the lack of reactivation was not due to genome loss during the culture period. To extend these results, we examined the ability of HCMVUL8 to reactivate in a humanized mouse model. In this model, CD34^+^ HPCs are engrafted into NOD-*scid* IL2Rγ_c_^null^ mice (huNSG) followed by HCMV infection (20). After viral latency is established, HCMV can be reactivated and disseminated into tissues through infected macrophages following treatment of mice with granulocyte colony-stimulating factor (G-CSF) and AMD3100 (20). As shown in Fig. 1D and E, only WT HCMV, but not HCMVΔUL8, showed increased DNA copy numbers in spleen and liver tissues, supporting the *in vitro* data. Together, these data demonstrate that UL8 is required for HCMV reactivation but not for establishment of latency both *in vitro* and *in vivo*.

**Fig 2:**
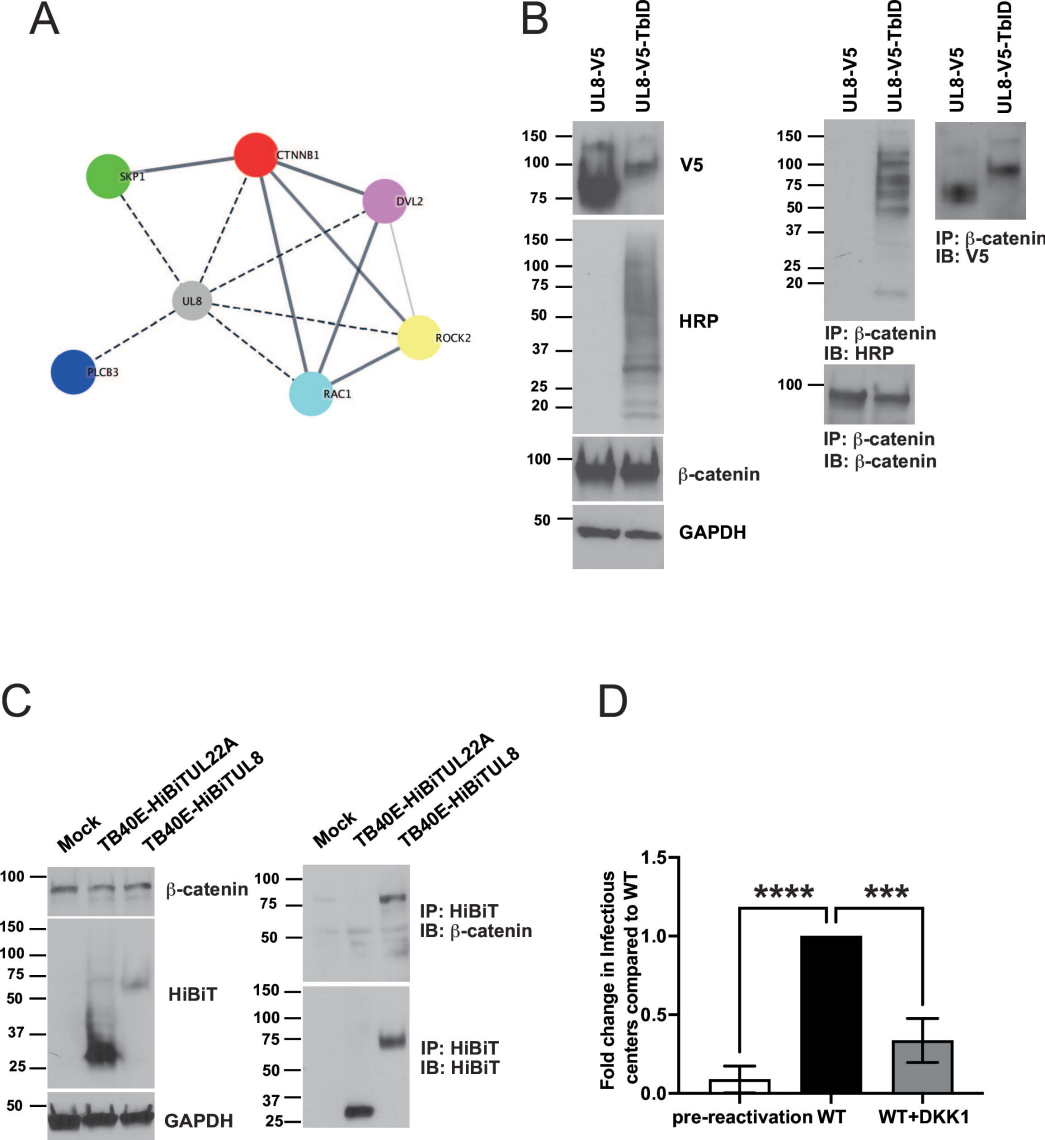
HCMV UL8 interacts with components of the Wnt/β-catenin pathway. (**A**) pUL8 interaction with components of the Wnt/β-catenin pathway (https://cytoscape.org) (21). Dashed lines indicate interactions identified by BioID-MS analysis, and solid lines represent protein-protein interactions derived from the STRING database (https://string-db.org). (**B**) HEK293 cells were transfected with plasmids expressing UL8-V5 or UL8-V5-TbID for 48 hours. Protein extracts were immunoprecipitated (IP) with β-catenin antibody and detected by Western blotting (IB) using HRP, V5, or β-catenin antibodies (right panel). A Western blot of total lysate is shown with GAPDH as a loading control (left panel). (**C**) phorbol 12-myristate 13-acetate (PMA)-treated THP-1 cells were infected with HCMV TB40/E encoding either HiBiT epitope-tagged UL22A (as negative control; HiBiTUL22A) or UL8 (HiBiTUL8) at an multiplicity of infection (MOI) of 3. HiBiT proteins were immunoprecipitated from lysates harvested at 72 hours post-infection (hpi) with Ms anti-HiBiT antibody and then separated by SDS-PAGE (right panel). HiBiT-tagged proteins and β-catenin were detected by Western blotting using NanoGlo HiBiT Blotting System and a rabbit β-catenin antibody. Blots of total cellular lysate are shown with GAPDH as a loading control (left panel). (**D**) Pure population of CD34^+^ HPCs infected with HCMV WT was isolated by FACS at 48 hpi and maintained in LTBMC medium. At 14 dpi, viable CD34^+^ HPCs were seeded onto fibroblast monolayers plated in 96-well dishes by limiting dilution in cytokine-rich media for reactivation in the presence or absence of DKK1 (50 ng/mL). Reactivation was scored as described above. Data shown in the fold change in reactivation normalized to HCMV WT post-reactivation. Bars represent the mean ± SEM of three independent experiments. Statistical significance was determined using one-way ANOVA with Tukey’s multiple comparison test (***P < 0.0005; ****P < 0.0001).

### The pUL8 cellular interactome

pUL8 is primarily a cell-associated membrane protein that has a distinctive cytoplasmic tail with two tyrosine-based endocytic motifs and a PDZ-binding domain (19). PDZ domains are protein interaction modules that often act as molecular scaffolds to recruit their binding partners and facilitate signaling (22). In order to characterize the proteins that are in proximity to UL8 (UL8 interactome), we used the proximity biotinylation (BioID system) method. The BioID system uses a promiscuous biotin ligase that biotinylates proteins within ~10 nm of the tagged protein (23, 24), making BioID-MS advantageous compared to IP-MS because this system captures not only strong and direct protein-protein interactions but also weak and/or transient interactions. Among biotin ligases, we used TurboID which catalyzes proximity labeling with great efficiency. To determine whether the C-terminal TurboID tag (TbID) alters pUL8 localization, we first performed immunofluorescence in cells transfected with pcDNA3.1-UL8-V5 or pcDNA3.1-UL8-V5-TbID. Consistent with a previous report (19), staining was abundant in the perinuclear region either in pcDNA3.1-UL8-V5 or pcDNA3.1-UL8-V5-TbID transfected HeLa cells, demonstrating that TurboID does not alter pUL8 localization (Fig. S3). Since UL8 is expressed at low levels during HCMV infection, we chose to perform the BioID-MS analysis in UL8V5-TbID-transfected HEK293 cells. Using the Significance Analysis of INTeractome algorithm (25, 26) for filtering specific protein interactions, 381 host proteins were predicted to be in close proximity to pUL8 (Data set S1). KEGG pathway analysis highlighted several components of the Wnt pathway (Fig. 2A), including the effector of the pathway β-catenin (CTNNB1), dishevelled homolog 2 (DVL2), ras-related C3 botulinum toxin substrate 1 (RAC1), Rho-associated protein kinase 2 (ROCK2), S-phase kinase-associated protein 1 (SKP1), and phospholipase C beta 3 (PLCB3) (FDR <0.1, Data set S1). To confirm these mass spectrometry results, we performed reciprocal immunoprecipitation (IP) using a β-catenin antibody. We were able to detect biotinylated proteins in cell extracts from HEK293 cells transfected with pcDNA3.1-UL8-V5-TbID but not in the control (Fig. 2B). As expected, UL8 was pulled down with β-catenin either in cells transfected with pcDNA3.1-UL8-V5-TbID or with pcDNA3.1-UL8-V5 (Fig. 2B).

In a recent publication on the HCMV interactome in fibroblasts infected with the Merlin strain, Nobre et al*.* identified β-catenin as potential UL8 interacting host protein (27). To verify that UL8 binds β-catenin in the context of infection and confirm that the binding is neither cell type nor viral strain specific, we infected differentiated THP-1 cells with the HCMV clinical strain TB40/E encoding for a HiBiT-tagged UL8 for 72 hours, and we then performed IP analysis. Similar to the report by Nobre et al. (27), β-catenin was immunoprecipitated in differentiated THP-1 cells infected with TB40/E-HiBiTUL8 (Fig. 2C). In contrast, the interaction was not detected in cells infected with a control virus, TB40/E-HiBiTUL22A, indicating that the binding to UL8 is specific.

To determine whether, like UL8, signaling through the Wnt/β-catenin pathway is required for efficient viral reactivation, we treated HCMV-latently infected CD34^+^ HPCs with the Wnt antagonist DKK-1. As shown in Fig. 2D, addition of DKK-1 at the time of reactivation significantly reduced the ability of the virus to fully reactivate (*P* = 0.0003), similar to ΔUL8-infected cells (Fig. 1B). Reduced viral reactivation in the presence of DKK-1 was not due to an inhibitory effect of the protein on HCMV lytic replication in fibroblasts or cytotoxicity in HPCs as demonstrated by the single and multistep step growth curves and by the cytotoxicity assay in CD34^+^ HPCs, respectively (Fig. S4).

Overall, these results provide evidence that HCMV requires an active Wnt/β-catenin pathway to efficiently reactivate from latency and suggest a key role for UL8 in the modulation of the pathway through the interaction with the effector β-catenin.

### UL8 and β-catenin interact with DVL2 via their PDZ-binding domain

**Fig 3:**
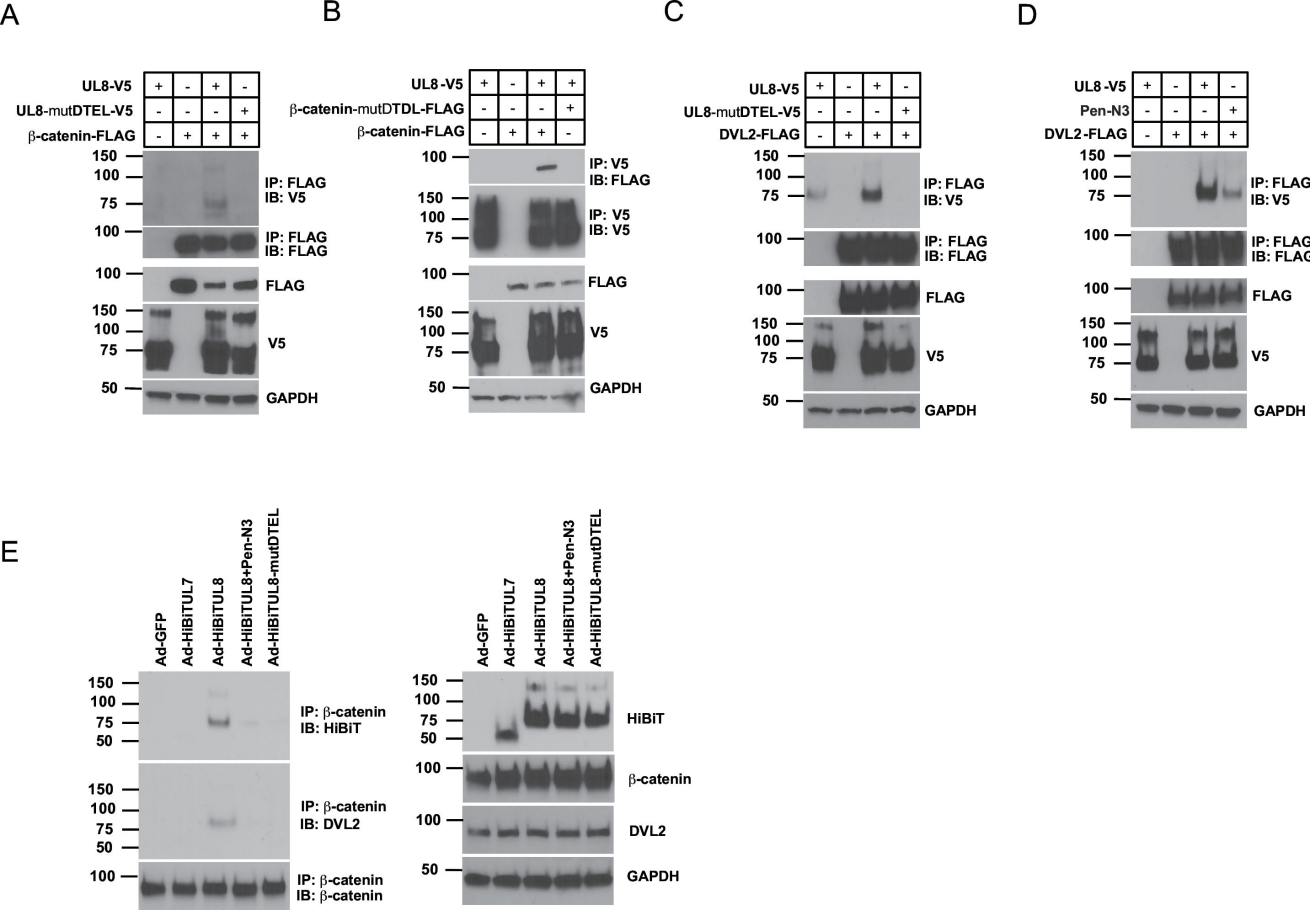
UL8 interacts with β-catenin and DVL2 through the PDZ domain. (**A**) HEK293 cells were transfected with plasmids expressing UL8-V5 or UL8-mutDTEL-V5 in which the DTEL sequence was mutated into four alanines in the presence of FLAG-tagged β-catenin. Using mouse anti-FLAG monoclonal antibody, UL8 was immunoprecipitated from cell lysate, and proteins, separated by SDS-PAGE. Immunoprecipitated proteins were detected by Western blotting using rabbit anti-V5 and anti-FLAG antibodies (upper panel). A Western blot of total lysate is shown with GAPDH as a loading control (lower panel). (**B**) HEK293 cells were transfected with plasmids expressing β-catenin-FLAG or β-catenin-mutDTDL-V5 in which the DTDL sequence was mutated into four alanines in the presence of UL8-V5. Using Ms anti-V5 monoclonal antibody β-catenin was immunoprecipitated from cell lysate, and proteins, separated by SDS-PAGE. Immunoprecipitated proteins were detected by Western blotting using rabbit anti-FLAG and anti-V5 antibodies (upper panel). A Western blot of total lysate is shown with GAPDH as a loading control (lower panel). (**C**) HEK293 cells were transfected with plasmids expressing UL8-V5 or UL8-mutDTEL-V5 in the presence of FLAG-tagged DVL2. Using Ms anti-FLAG monoclonal antibody, UL8 was immunoprecipitated from cell lysate, and proteins, separated by SDS-PAGE. Immunoprecipitated proteins were detected by Western blotting using rabbit anti-V5 and anti-FLAG antibodies (upper panel). A Western blot of total lysate is shown with GAPDH as a loading control (lower panel). (**D**) HEK293 cells were transfected with plasmids expressing UL8-V5 or DVL2-FLAG and then treated with DMSO or Pen-N3 peptide (10 μM). Using mouse anti-FLAG monoclonal antibody, UL8 was immunoprecipitated from cell lysate, and proteins, separated by SDS-PAGE. Immunoprecipitated proteins were detected by Western blotting using rabbit anti-V5 and anti-FLAG antibodies (upper panel). A Western blot of total lysate is shown with GAPDH as a loading control (lower panel). (**E**) Neonatal human dermal fibroblasts (NHDFs) were transduced with adenoviral constructs expressing GFP, UL7, or UL8-mutDTEL or transduced with AdUL8 and then treated with DMSO or Pen-N3 peptide (10 μM) for 48 h. Using mouse anti-β-catenin monoclonal antibody, UL8 and DVL2 were immunoprecipitated from cell lysate, and proteins, separated by SDS-PAGE. Immunoprecipitated proteins were detected by Western blotting using NanoGlo HiBiT Blotting System and a rabbit DVL-2 antibody (left panel). A Western blot of total lysate is shown with GAPDH as a loading control (right panel).

The pUL8 22-aa cytoplasmic tail has a PDZ-binding domain [DTEL (aa 321 to 324aa)] completely conserved among all strains of HCMV (19). β-Catenin has also a PDZ-binding domain that recognizes the dishevelled homolog 1 (DVL1) PDZ domain, and the interaction between the two proteins is required to induce nuclear accumulation of β-catenin (28). Therefore, we first addressed whether the UL8 and β-catenin PDZ-binding domains are required for their interaction. HEK293 cells were co-transfected with a plasmid expressing FLAG tagged β-catenin and UL8-V5 or UL8-mutDTEL-V5 in which the PDZ-binding sequence was mutated into four alanine residues (DTEL>AAAA). As shown in Fig. 3A, β-catenin and UL8 co-immunoprecipitated only when UL8 has an intact PDZ-binding domain. In agreement with this, mutagenesis of the β-catenin PDZ-binding domain abrogates the interaction with UL8 (Fig. 3B). The BioID-MS analysis showed that UL8 is proximal to DVL2, which contains a PDZ domain. To evaluate if there was a direct binding between the two proteins, we performed co-immunoprecipitation in HEK293 transfected with a FLAG-tagged DVL2 plasmid and UL8-V5 or UL8-mutDTEL-V5. As shown in Fig. 3C, UL8 binds DVL2 only when UL8 PDZ-binding domain is functional. To additionally confirm the interaction between UL8 and DVL2, we used a peptide, Pen-N3, that specifically blocks interactions with the PDZ domain of DVL1, DVL2, and DVL3 (29). When cells were co-transfected with DVL2-FLAG and UL8-V5 plasmids and then treated with Pen-N3 peptide or DMSO, we observed a reduction in binding of UL8 to DVL2 when treated with Pen-N3 compared to cells treated with DMSO (Fig. 3D). These co-IP experiments demonstrate that the UL8 PDZ-binding domain is essential for the interaction with β-catenin and DVL2. We then investigated if β-catenin and DVL2 form a stable complex with UL8 since only DVL2 contain a PDZ domain while UL8 and β-catenin encode PDZ-binding sequences. Human dermal fibroblasts were transduced with adenoviral constructs expressing GFP, UL7, UL8, or UL8-mutDTEL or transduced with AdUL8 and then treated with Pen-N3 peptide or DMSO and immunoprecipitated with a β-catenin antibody. As shown in Fig. 3E, UL8 and DVL2 immunoprecipitated with β-catenin only in cells transduced with UL8 and not the controls GFP or UL7. Moreover, mutagenesis of the UL8 PDZ-binding domain or treatment with Pen-N3 peptide prevented the formation of the β-catenin-UL8-DVL2 complex.

These results indicate that UL8 and β-catenin form a complex with DVL2 through their PDZ-binding domains and that the Pen-N3 peptide can efficiently target and disrupt these interactions.

### Interaction between UL8 and β-catenin promotes stabilization and transcriptional activation

**Fig 4:**
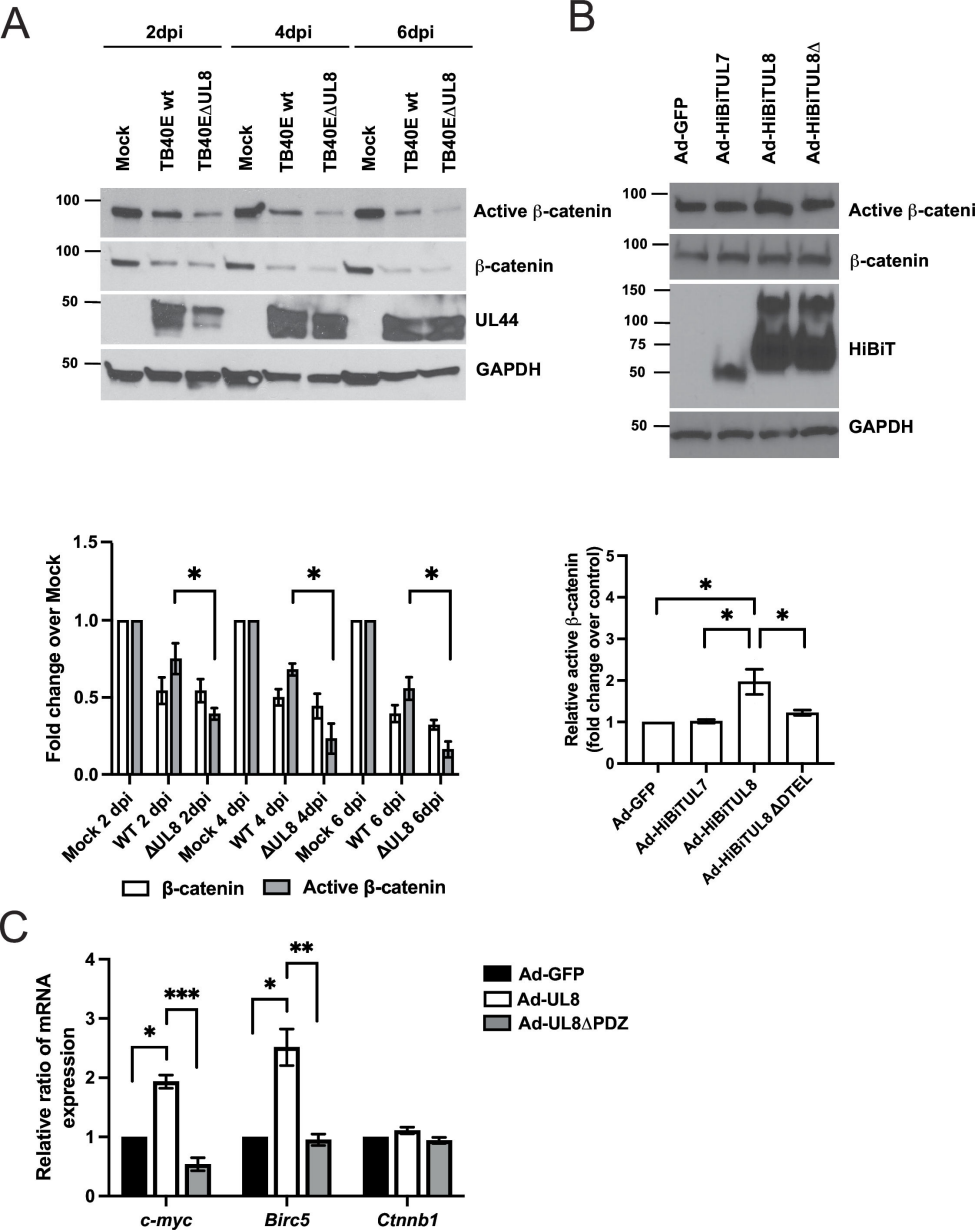
UL8 is required for the stabilization of β-catenin. (**A**) NHDFs were infected with HCMV TB40/E WT or ΔUL8 at an MOI of 1. At the indicated times post-infection, protein lysates were generated and immunoblotted for active (unphosphorylated) β-catenin, total β-catenin, HCMV UL44, and GAPDH as loading control. Relative protein expression of three independent Western blotting was quantified using Image J. Values are means ± standard error of the means (SEM) (error bars). Statistical significance was determined by unpaired Student’s *t*-test. *P < 0.05 compared to active-β-catenin in WT-infected cells at each time point. (**B**) NHDFs were transduced with Ad-GFP, -UL7, -UL8, or -UL8ΔPDZ for 48 hours at MOI of 500. Protein lysates were generated and immunoblotted for active-β-catenin, β-catenin, HiBiT, and GAPDH as loading control. Relative protein expression of three independent Western blotting was quantified using Image J. Values are means ± standard error of the means (SEM) (error bars). Statistical significance was determined using one-way ANOVA with Tukey’s multiple comparison test. *P < 0.05 compared to transduced with Ad-GFP. (**C**) CD34^+^ HPCs were transduced at an MOI of 125 with Ad-GFP, -UL8, or -UL8-mutDTEL for 48 h and then FACS isolated for viable CD34^+^ GFP^+^ HPCs. RNA was isolated using Trizol, and qRT-PCR for *c-myc*, *Birc5*, and *Ctnnb1* was performed. Values are means ± standard error of the means (SEM) (error bars) compared to Ad-GFP for three independent experiments. Statistical significance was determined using one-way ANOVA with Tukey’s multiple comparison test (*P < 0.05, **P < 0.005; ***P < 0.0005).

Previous studies on HCMV and β-catenin have shown that during lytic replication, levels of β-catenin that are membrane-associated, in cytosolic pools, and in nuclear fractions, are reduced after infection (30). Conversely, inhibition of the Wnt pathway is associated with impaired HCMV replication (31), suggesting a very fine balance between virus and β-catenin levels that can either maintain or abrogate lytic replication. To address the impact of UL8 on β-catenin levels, we performed a time course in human fibroblasts infected with HCMV WT or ΔUL8 virus. As shown in Fig. 4A, HCMV infection decreases the pool of total β-catenin throughout the time course without significant differences between WT and ΔUL8 infection. Interestingly, levels of active (unphosphorylated) β-catenin are lower in ΔUL8-infected cells compared to WT, supporting a role of UL8 in β-catenin stabilization. Comparable levels of HCMV UL44 were observed in the WT- and ΔUL8-infected cells at each time point ruling out that the differences in active β-catenin levels were due to discrepancies in replication between the two viruses.

We next investigated if UL8 had an impact on active β-catenin levels outside of the context of HCMV infection. As shown in Fig. 4B, cells transduced with an adenovirus expressing UL8 have higher levels of active β-catenin compared to GFP (*P* = 0.0103) and UL7 (*P* = 0.0114) controls. Moreover, interaction between UL8 and β-catenin is required to promote stabilization of the protein as demonstrated by the decrease levels of active β-catenin in cells transduced with the UL8-mutDTEL construct compared to full length UL8 (*P* = 0.0403).

To test whether increased levels of active β-catenin had functional consequences, we examined expression of Wnt target genes *c-myc* and survivin (*Birc5*). We transduced CD34^+^ HPCs with Ad-GFP, Ad-UL8, or Ad-UL8-mutDTEL and FACS isolated a pure population of viable CD34^+^ GFP^+^ cells to determine the effect of UL8 on *c-myc* and *Birc5* mRNA levels. As shown in Fig. 4C, upon UL8 expression *c-myc* and *Birc5* mRNA levels were increased compared to the control GFP (*P* = 0.0023; *P* = 0.0144). Mutagenesis of the PDZ-binding domain significantly decreased UL8-mediated transcriptional activation of *c-myc* (*P* = 0.0002) and *Birc5* (*P* = 0.0077). No significant difference was found between β-catenin (*Ctnnb1*) mRNA levels in CD34^+^ HPCs transduced with Ad-UL8 or Ad-UL8-mutDTEL compared to Ad-GFP, indicating that regulation of β-catenin occurred at the protein, rather than at the transcriptional level. Importantly, a similar change in transcriptional levels of *c-myc* and *Birc5* was observed in CD34^+^ HPCs stimulated with Wnt3A (Fig. S5), suggesting that the stabilization of β-catenin by UL8 in CD34^+^ HPCs is functionally relevant.

Taken together, these data indicate that interaction between β-catenin and UL8 promotes stabilization and transcriptional activation of β-catenin-dependent genes in CD34^+^ HPCs.

### Interaction among UL8, β-catenin, and DVL2 is required for efficient viral reactivation

**Fig 5:**
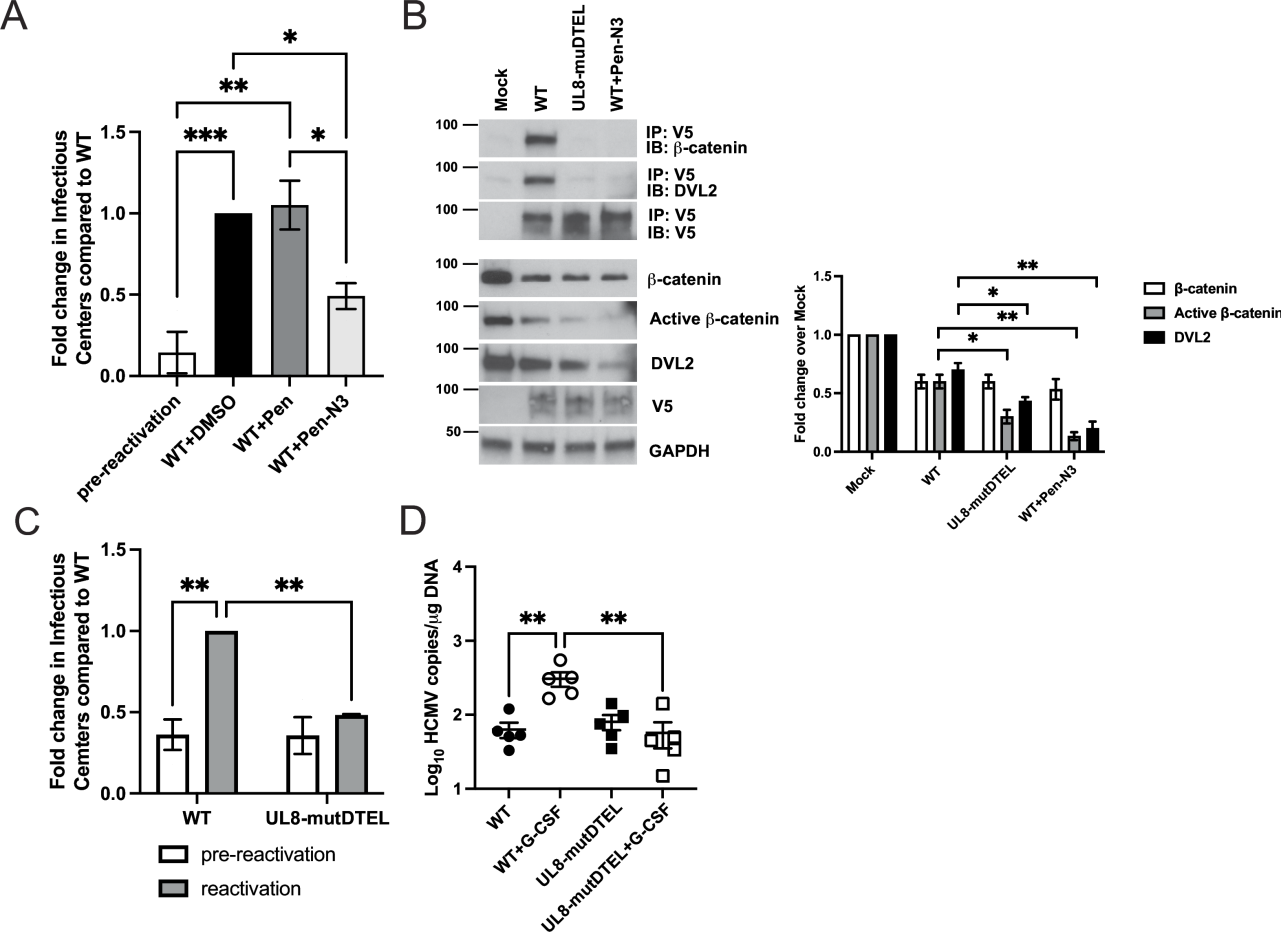
UL8-β-catenin-DVL2 complex is required for HCMV reactivation. (**A**) Pure population of CD34^+^ HPCs infected with HCMV WT was isolated by FACS at 48 hours post-infection (hpi) and maintained in LTBMC medium. At 14 dpi, viable CD34^+^ HPCs were seeded onto fibroblast monolayers plated in 96-well dishes by limiting dilution in cytokine-rich media for reactivation in the presence of Pen-N3, Pen (10 µM), or DMSO. Reactivation was scored as described above. Data shown in the fold change in reactivation normalized to HCMV WT post-reactivation. Bars represent the mean ± SEM of three independent experiments. Statistical significance was determined using one-way ANOVA with Tukey’s multiple comparison test (*P < 0.05*;* **P < 0.005*;* ***P < 0.0005). (**B**) NHDFs were mock infected or infected with TB40/E UL8-V5 (WT) and then treated with Pen-N3 or DMSO or infected with TB40/E UL8-mutDTEL-V5 (UL8-mutDTEL) at an MOI of 1 for 72 hours. Using mouse anti-V5 antibody, β-catenin and DVL2 were immunoprecipitated from cell lysate, and proteins, separated by SDS-PAGE. Immunoprecipitated proteins were detected by Western blotting using rabbit anti-β-catenin and mouse anti-DVL2 antibody (upper panel). Blots of total cellular lysate are shown with GAPDH as a loading control (lower panel). Right, relative protein expression of three independent Western blotting was quantified using Image J. Values are means ± standard error of the means (SEM) (error bars). Statistical significance was determined by unpaired Student’s *t*-test. *P < 0.05; **P < 0.005 compared to active-β-catenin and DVL2 in WT-infected cells. (**C**) Pure population of CD34^+^ HPCs infected with TB40/E UL8-V5 (WT) or TB40/E UL8-mutDTEL-V5 (UL8-mutDTEL) were isolated by FACS at 48 hpi and maintained in LTBMC medium. Latency and reactivation assays were performed as described above. Bars represent the mean ± SEM of three independent experiments. Statistical significance was determined using two-way ANOVA with Tukey’s multiple comparison test (*P < 0.05*;* **P < 0.005). (**D**) Sublethally irradiated NOD-*scid* IL2Rγ_c_^null^ mice were engrafted with CD34^+^ HPCs (huNSG) and subsequently injected with human fibroblasts previously infected with HCMV WT or UL8-mutDTEL mutant as indicated. At 4 weeks post-infection, viral reactivation was triggered with G-CSF and AMD-3100 and HCMV genome quantified as described above.

Finally, we wanted to address whether the UL8-β-catenin-DVL2 complex was required for viral reactivation. Based on the results from the β-catenin, UL8, and DVL2 pulldown (Fig. 3E), we first treated HCMV latently infected CD34^+^ HPCs with Pen-N3 peptide at the time of reactivation. As shown in Fig. 5A, addition of Pen-N3 but not the control peptide Pen at the time of reactivation significantly (*P* = 0.0131) decreased the ability of the virus to reactivate. The reduction in reactivation was not due to an inhibitory effect of the peptide on lytic replication or a cytotoxic effect as demonstrated by the single and multistep growth curves and cytotoxicity assay (Fig. S6). We then generated a UL8 mutant virus in which the DTEL sequence was mutated into four alanine residues (DTEL>AAAA), and a V5-tag was added at the C-terminus (UL8-mutDTEL). We first performed UL8 pulldowns in fibroblasts infected with WT and then treated with Pen-N3 or DMSO or UL8-mutDTEL mutant virus. As shown in Fig. 5B, β-catenin and DVL2 were immunoprecipitated only in WT-infected cells but not in cells infected and treated with Pen-N3 or infected with UL8-mutDTEL mutant. Interestingly, we observed less active β-catenin in cells infected with the UL8-mutDTEL mutant compared to WT (*P* = 0.02) and decreased levels of DVL2 (*P* = 0.01). Treatment with Pen-N3 peptide also reduced active β-catenin and DVL2 levels compared to WT (*P* = 0.002; *P* = 0.003). Lastly, we performed latency and reactivation assays in CD34^+^ HPCs infected with WT or UL8-mutDTEL virus *in vitro* and *in vivo*. As shown in Fig. 5C and Fig. S6, mutagenesis of UL8 PDZ-binding domain impaired reactivation (*P* = 0.0047) similarly to the Pen-N3 peptide. Analysis of UL8-mutDTEL growth curve (Fig. S8) and genomes (Fig. S7) demonstrated that the lack in reactivation was not due to reduced growth capacity of the mutant compared to WT or inability to retain the genome during latency. Furthermore, infection of huNSG mice with UL8-mutDTEL virus resulted in reduced levels of viral DNA (*P* = 0.0025) in the spleen of the animals treated with G-CSF and AMD3100 compared to WT (Fig. 5D), indicating that the UL8-mutDTEL was unable to reactivate.

**Fig 6:**
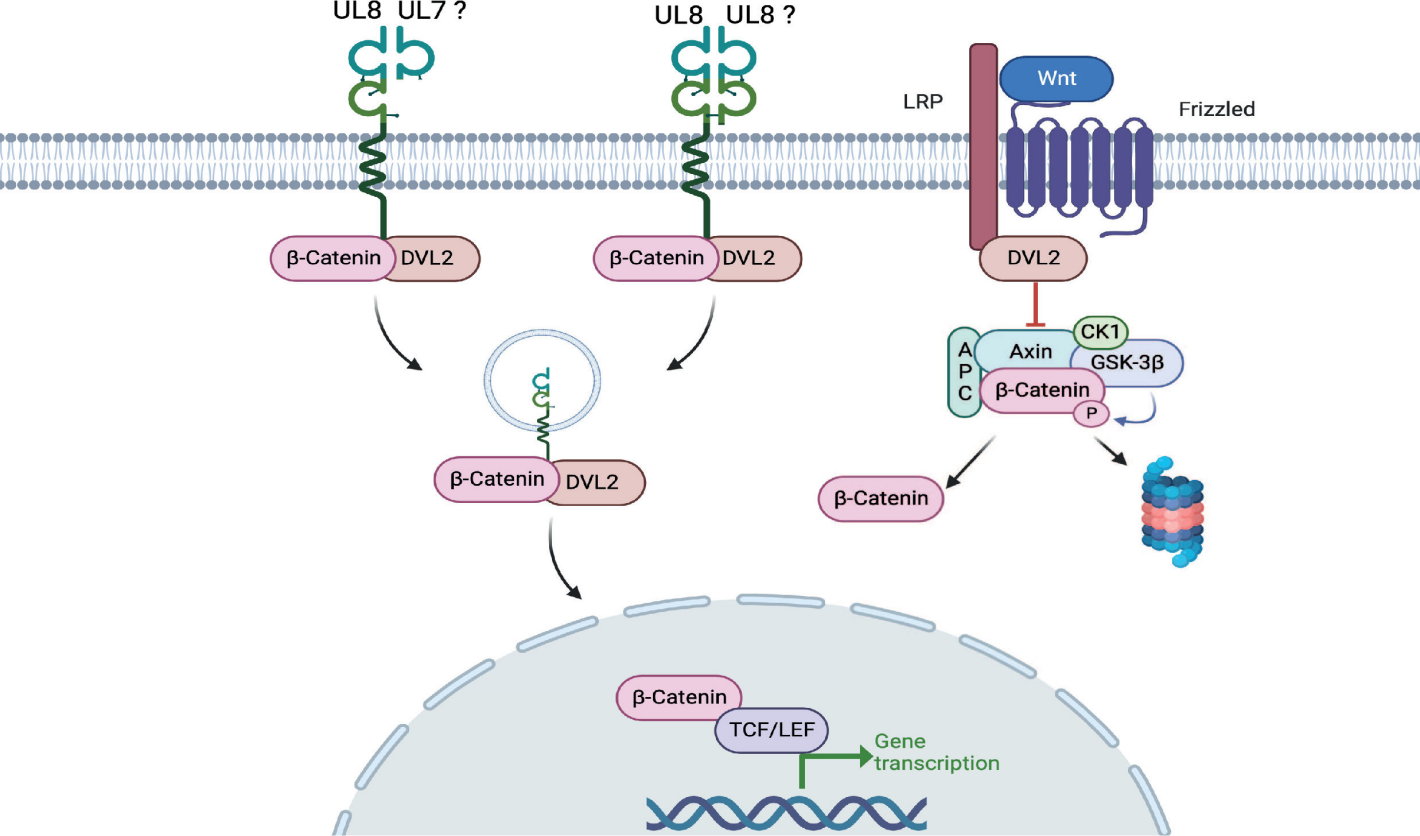
Model of UL8-mediated stabilization of β-catenin signaling pathway. The right side of the model represents components of the Wnt/Frizzled-mediated activation of β-catenin. In the off state, β-catenin is localized in the cytoplasm bound to a multiprotein proteasomal destruction complex (Axin, APC) that promotes GSK3β-and casein kinase 1 (CK1)-mediated β-catenin phosphorylation and degradation. In the on state, the destruction complex is disrupted in a Dishevelled (DVL)-mediated way upon Wnt binding to the Frizzled/LPR, and β-catenin can translocate into the nucleus. Activation of UL8 by secreted UL7, UL8 itself, or a currently unknown factor bypasses Wnt receptor activation of β-catenin signaling through recruitment of β-catenin and DVL2 to the plasma membrane promoting endocytic uptake, β-catenin stabilization, and transcriptional activation. This figure was created using BioRender.

All together, these results demonstrate that UL8 forms a stable complex with β-catenin and DVL2 that enhances β-catenin-mediated gene expression, and these interactions are essential for efficient viral reactivation *in vitro* and *in vivo*.

## DISCUSSION

In this study, we demonstrated for the first time that activation of Wnt/β-catenin signaling by UL8 is important for reactivation of HCMV in CD34^+^ HPCs. Indeed, we found that deletion of UL8 impairs viral reactivation *in vitro* and *in vivo.* Using the proximity biotinylation (BioID system) approach, we discovered that UL8 is in proximity to several proteins in the Wnt signaling pathway, including β-catenin and DVL2. Immunoprecipitation analysis verified the interaction between UL8, β-catenin, and DVL2. In addition, we have shown that UL8 is required for β-catenin stabilization and transcriptional activation. Finally, we observed that the binding of UL8 and β-catenin to the DVL2 PDZ domain is required for efficient viral reactivation. Taken together, HCMV manipulates the Wnt/β-catenin pathway for efficient viral reactivation, and UL8 plays a critical role in the stabilization and activation of the effector of the pathway, β-catenin.

Mounting evidence links viral infections to β-catenin activation and pathogenesis. For instance, the human papillomavirus (HPV) E6 and E7 oncogenes appear to contribute to activation of β-catenin signaling in HPV16-positive oropharyngeal squamous carcinoma cells, the hepatitis C virus (HCV)-encoded core protein potentiates Wnt/β-catenin signaling in hepatocellular carcinoma cells (32, 33), and pharmacological activation of the β-catenin/TCF1 pathway with glycogen synthase kinase-3 (GSK-3) inhibitors reactivates latent HIV-1 in CD4^+^ T cells (34). Using the BioID system, we identified HCMV UL8 as a new viral regulator of the Wnt/β-catenin pathway (Fig. 2B), and UL8 is able to form a complex with β-catenin and DVL2 via the PDZ-binding domain (Fig. 3E). The formation of this complex leads to β-catenin stabilization (Fig. 4A and B) and transcriptional activation of the β-catenin-dependent genes *c-myc* and *Birc5* (Fig. 4C).

Among α- and γ-herpesvirus, several viral proteins are known to interact with components of the Wnt pathway to control latency (12 – 14, 16). For example, in Bovine herpesvirus 1 (BoHV-1) infection, β-catenin is readily detected in a subset of latently infected trigeminal ganglia neurons but not in uninfected cells or during reactivation from latency, suggesting that, in this case, an active Wnt pathway promotes latency (15). Moreover, the Epstein-Barr virus (EBV) latent membrane protein 2A that is involved in latency maintenance in EBV-infected B lymphocytes was found to inactivate GSK3β and stabilize β-catenin (12). In contrast, we found that, in HCMV, stabilization of β-catenin by UL8 is required for viral reactivation (Fig. 1B, D, E and 5C, D). Interestingly, US28, an HCMV-encoded chemokine receptor essential for viral reactivation *in vitro* and *in vivo* (35), has been shown to activate β-catenin via a non-conventional pathway, involving Rho kinase (36). Moreover, US28 expression in intestinal epithelial cells (IECs) promotes activation of Wnt signaling leading to accumulation of β-catenin, expression of Wnt target genes, and proliferation of IECs (37). Finally, Ueland et al*.* demonstrated that low levels of DKK1 in solid organ transplant recipients are predictors of treatment failure and viral reactivation (38). Together with our findings that treatment of latently HCMV infected CD34^+^ HPCs at the time of reactivation with DKK1 decreases the ability of the virus to reactivate *in vitro* (Fig. 2D), this suggests a conserved role of the Wnt pathway in CMV infection that is different from α- and γ-herpesvirus.

A recent publication by Nobre et al*.* identified β-catenin as potential UL8 interactor during HCMV infection in fibroblasts with the Merlin strain (27). Here, we demonstrated that UL8 immunoprecipitates with β-catenin in differentiated THP-1 cells infected with HCMV TB40/E (Fig. 2C). The UL8 gene encodes a type I transmembrane protein comprising a signal peptide, an Ig-V-like domain, a stalk, a transmembrane region, and a cytoplasmic tail. The degree of amino acid sequence identity of UL8 found among different HCMV strains is between 79.9% and 100% (19). Interestingly, the UL8 cytoplasmic tail, which has two tyrosine-based endocytic motifs and a PDZ-binding domain, is completely conserved among all strains. It has been shown that mutation of the UL8 endocytic motifs (Y305/314A) significantly reduces UL8 internalization; however, the functionality of the PDZ has never been shown (19). PDZ domains are protein interaction modules that often recognize short amino acid motifs at the C-terminus of target proteins (39). For example, β-catenin has been shown to interact with PDZ-domain-containing proteins through its C terminus, including DVL1 (28, 40). Furthermore, Zhang et al. reported inhibition of Wnt signaling by Dishevelled PDZ peptides (29). Our data demonstrate, for the first time, that UL8 PDZ-binding domain is essential for the interaction with β-catenin and DVL2 (Fig. 3A through D) and the DVL-PDZ peptide Pen-N3 is able to prevent not only the formation of the UL8-β-catenin-DVL2 complex (Fig. 3E and 5B) but also HCMV reactivation (Fig. 5A).

As we discussed in our previous publication (18), HCMV reactivation is a multistep process that involves significant changes in the intracellular environment. We have previously demonstrated that secretion of pUL7 and binding to the Flt-3R is important for myeloid cell differentiation and viral reactivation (18). We now have evidence that a closely related protein UL8 is also required for reactivation by promoting signaling through the Wnt/β-catenin pathway. UL8 most likely functions as a receptor, and only a small fraction is proteolytically cleaved from the surface (19). Interestingly, there is accumulating evidence that endocytosis of the Wnt ligand-receptor complex facilitates Wnt/β-catenin signaling (41). Moreover, signaling amplification involves endosome-based delivery of activated receptors into the perinuclear region of the cell (20, 42 – 44). Therefore, our working model is that activation of UL8 by secreted UL7, UL8 itself (27), or a currently unknown factor bypasses Wnt receptor activation of β-catenin signaling through recruitment of β-catenin and DVL2 to the plasma membrane and endocytic uptake that protects β-catenin from degradation and supports transcriptional activation and ultimately viral reactivation (Fig. 6).

Knowledge from our study, together with the progress in the development of Wnt-signaling inhibitors for cancer treatment, will open a new avenue for targeted HCMV therapies and innovative clinical interventions that will improve the overall survival of transplant patients.

## MATERIALS AND METHODS

### Cells and reagents

Feeder-free hESC were obtained from WiCell [WA01/H1, hPSCReg identifier (ID) WAe001-A, NIH approval no. NIHhESC-10-0043]. Cells were thawed and plated on Matrigel-coated six-well plates in complete mTeSR1 (Stem Cell Technologies). CD34^+^ HPCs were differentiated using a commercial feeder-free hematopoietic differentiation kit (STEMdiff Hematopoietic Kit; Stem Cell Technologies) as previously described (45). Neonatal human dermal fibroblasts (NHDFs) from ATCC were cultured with Dulbecco’s modified Eagle’s medium (DMEM) (Cellgro) supplemented with 10% fetal bovine serum (FBS) (HyClone, Thermo Scientific), 1 mM sodium pyruvate, 2 mM glutamax, and 100 U/mL pen/strep. Human Embryonic Kidney 293 cell line (HEK293) (Microbix Biosystems, Inc.) was cultured in Minimum Essential Medium (Cellgro) supplemented with 10% FBS, 1 mM sodium pyruvate, 2 mM glutamax, and 100 U/mL pen/strep. The M2-10B4 murine stromal cell line expressing human interleukin-3 (IL-3) and G-CSF and the S1/S1 murine stromal cell line expressing human IL-3 and stem cell factor were obtained from Stem Cell Technologies. THP-1 cells from ATCC were cultured in Roswell Park Memorial Institute (RPMI) medium supplemented with 10% FBS, 1 mM sodium pyruvate, 2 mM glutamax, 50 µM β-mercaptoethanol, and 100 U/mL pen/strep. Cells were treated with 100 nM 12-o-tetradecanoylphorbol-13-acetate and plated on tissue culture-treated plates to promote monocyte-to-macrophage differentiation. Recombinant human DKK1 protein was purchased at R&D System; the Dvl-PDZ domain inhibitor, Peptide Pen-N3, at MilliporeSigma; and the control peptide Antennapedia (Pen), at Eurogentec.

### Plasmids

UL7-8 from TB40E (EF999921) containing a HiBiT sequence (5′-gtg agc ggc tgg cgg ctg ttc aag aag att agc-3′) after the signal peptide, HiBiT-UL8-V5, HiBiT-UL8-mutDTEL-V5, where amino acids Asp-Thr-Glu-Leu were substituted to Ala-Ala-Ala-Ala, UL8-V5-TbID (24) inserts were synthesized and cloned into pcDNA3.1 at GenScript. Human beta-catenin pcDNA3 was a gift from Eric Fearon (Addgene plasmid #16828) (46), and 3XFlagDVL2 was a gift from Jeff Wrana (Addgene plasmid #24802) (47). pCDNA3-β-catenin-mutDTDL-Flag, in which amino acids Asp-Thr-Asp-Leu were substituted to Ala-Ala-Ala-Ala, was synthesized at GenScript. UL22A from TB40E (EF999921) containing a HiBiT sequence after the signal peptide and a FLAG-tag at the C-terminus was synthesized by Thermo Fisher. The pAdTrack-HiBiTUL7, pAdTrack-HiBiTUL8V5, and pAdTrack-HiBiTUL8V5-mutDTEL were generated by subcloning the inserts from pCDNA3.1 into pGEM-T easy vector system and then into pAdTrack as previously described (48).

### Proximity biotinylation

HEK293 cells were transfected with pDNA3.1-UL8-V5 or pDNA3.1-UL8-V5-TbID for 48 hours with Lipofectamine 2000, and then, 50 µM of biotin was added to the media for 6 hours. Cells were lysed in lysis buffer containing 50 mM Tris-HCl, pH 8, 1% Nonidet P-40, 150 mM NaCl, and 1× protease inhibitor cocktail (P8340, Sigma), 1× phosphatase inhibitor cocktail 1 (P2850; Sigma), 1× phosphatase inhibitor cocktail 2 (P5726; Sigma), and insoluble debris were pelleted at 10,000 × *g* for 10 min at 4°C. One milligram of protein lysates was incubated at 4°C overnight with 500 µL of streptavidin conjugated to magnetic beads (New England BioLabs, Ipswich, MA, USA). Beads were washed once in 1.5 mL of wash buffer 1 (2% SDS in H_2_O), once with wash buffer 2 [0.1% deoxycholate, 1% Triton X-100, 500 mM NaCl, 1 mM EDTA, and 50 mM HEPES (pH 7.5)], once with wash buffer 3 [250 mM LiCl, 0.5% NP-40, 0.5% deoxycholate, 1 mM EDTA, 10 mM Tris (pH 8.1)], and then twice with wash buffer 4 [50 mM Tris (pH 7.4), 50 mM NaCl]. To evaluate sample integrity, 10% of the total was retained for immunoblots. The remaining beads were spun at 2,000 × *g* and resuspended in 50 µL of 50 mM ammonium bicarbonate for mass spectrometry.

### HCMV mutant viruses

The HCMV TB40/E bacterial artificial chromosome (BAC) was previously engineered to express green fluorescent protein (GFP) as a visual marker of infection (49). The TB40/E-HiBiTUL8, TB40/E-UL8-V5, TB40/E-UL8-mutDTEL-V5, and TB40/E-HiBiTUL22A-FLAG were constructed using the Galk recombineering method (50). In the first step, the *Galk/kan* ORF was amplified from pYD-C255, a GalK-kan (galactokinase-kanamycin) dual-expression plasmid (a gift from Dr. Yu, Washington University in St. Louis), using the primers shown in Table S1. To replace the *Galk/Kan* cassette in the second step, HiBiTUL8, UL8-V5, and HiBiTUL22A were amplified from pcDNA3-HiBiTUL7-8, pcDNA3-UL8-V5-TbID, and pMS-HiBitUL22A-FLAG plasmids using the primers shown in Table S1. TB40E-UL8-mutDTEL-V5 was generated by inserting the *Galk/kan* cassette at 3′ end of UL8 ORF, and in the second step, we used oligonucleotides (Table S1) with overlap to UL8-mutDTEL-V5 to substitute amino acids Asp-Thr-Asp-Leu to Ala-Ala-Ala-Ala. To construct a TB40/E based mutant deleted for UL8, we performed en passant recombination in *E. coli* strain GS1783 (51). Briefly, we amplified a recombination insert consisting of an *I-SceI* recognition sequence followed by an aminoglycoside 3′-phosphotransferase (kanamycin resistance, KanR) selection marker with primers shown in Table S1. Hence, 50 bp upstream of the UL7 termination codon was repeated in both primers, creating a 50-bp long repeated region flanking the recombination insert. The PCR product generated to replace exon 2 of ORF UL8 was inserted into the TB40 BAC backbone by homologous recombination through heat shock induction of the λ phage-derived Red recombination genes in *E. coli* strain GS1783. Recombinants were selected on kanamycin containing agar plates, and positive clones were screened by *HindIII* digest and Sanger sequences across the altered genomic locus to control from genome integrity and to select for clones that contained the desired genomic configuration. The KanR selection maker was removed seamlessly by homologous recombination through the arabinose-induced induction of *I-SceI* expression resulting in a DNA double-strand break at the introduced *I-SceI* recognition sequence in the BAC allowing the artificially created 50-bp-long repeat regions to overlap and recombine after heat shock induction of the λ phage-derived Red recombination genes in GS1783. Clones were screened for the absence of Galk or KanR, and negative clones were analyzed by *HindIII* restriction digest analysis and Sanger sequencing. The final BAC clones were furthermore analyzed by next-generation sequencing using an Illumina iSeq sequencer to exclude off-target mutations. Virus was reconstituted by transfecting the BAC genomes into NHDF. Plaque purification was performed, and viral stocks and titers were generated as previously described (18).

### qRT-PCR

Total RNA was isolated from infected, transfected, or treated cells using the Trizol RNA isolation method following the manufacturer’s directions. cDNA was prepared using 10 to 1,000 ng of total RNA and random hexamer primers. Samples were incubated at 16°C for 30 min, 42°C for 30 min, and 85°C for 5 min. Real-time PCR (Taqman) was used to analyze cDNA levels in transfected or infected samples. An ABI StepOnePlus Real Time PCR machine was used with the following program for 40 cycles: 95°C for 15 s and 60°C for 1 min. *c-myc*, *Birc5*, *Ctnnb1*, and *GAPDH* primer/probe sets were obtained from Thermo Fisher Scientific. Relative expression was determined using the ΔΔCt method using 18S as the standard control. For UL7 expression, we used the following set of primers and probe: UL7_F primer 5′-ACTACGTGTCGTCGCTGGATT-3′; UL7_R primer 5′-ACAACTTCCACCACCCCATAAT; UL7 probe 6FAM-CATGGCCTTGGTAGGTG-MGBNFQ. For UL8 expression, we used the following set of primers and probe: UL8_F primer 5′-TCACGGAAACCGCCAATAC-3′; UL8_R primer 5′-AACGATAATCAGCATCCAAGAG-3′; UL8 probe 6FAM-TTCGGCSSCGCAACTACGGTTATTCCAC-TAMRA.

### Adenoviruses

pAdTrack-CMV (Addgene plasmid #16405) and AdEasier-1 cells (Addgene #16399) were gifts from Bert Vogelstein (52). The pAdTrack plasmids were linearized by digesting with restriction endonuclease *Pme I* and subsequently recombined into *E. coli* BJ5183 cells containing the adenoviral backbone plasmid pAdEasy-1 (AdEasier-1 cells). Recombinants were selected for kanamycin resistance, and the recombination confirmed by restriction endonuclease analyses. Finally, the recombinant plasmids were linearized with *PacI* before transfection with Lipofectamine 2000 (Thermo Fisher) into the adenovirus packaging cell lines HEK293 cells. The control vector Ad-GFP, Ad-HiBiTUL7, Ad-HiBiTUL8-V5, and Ad-HiBiTUL8-mutDTEL-V5 were produced, purified, and titered in HEK293, as previously described (48).

### Adenovirus transduction of CD34^+^ HPCs

hESC-derived CD34^+^ HPCs were resuspended at low volume in Iscove's modified Dulbecco's medium (IMDM) containing 10% BIT serum supplement, L-glutamine, low-density lipoproteins, B-ME, and stem cell cytokines as previously described (48) in a low binding 24-well plate (Corning low-attachment Hydrocell). Cells were then infected with adenoviruses at an MOI of 125 for 4 hours with continual rocking, then spin infected at 300 g for 30 min, resuspended, and cultured overnight. Culture conditions were supplemented with additional media, and infection continued for a total of 48 hours. Samples were then FACS isolated for pure populations of transduced HPCs (viable, CD34^+^, GFP^+^) as previously described (18).

### Immunoprecipitation

Lysates were prepared from transfected, adenoviral transduced, or HCMV infected cells as described above. Soluble proteins were quantified with a bicinchoninic acid assay (Thermo Fisher), and equal quantities of protein were used for immunoprecipitation. Immunoprecipitation was performed by incubating 300–500 μg of protein lysate with 1:100 of primary antibody for 3 h at 4°C, followed by capture of antigen-antibody complexes with 20 µL of protein A or G magnetic beads (Cell Signaling). The resin beads were then rapidly washed five times with Tris-buffered saline buffer with 0.1% Nonidet P-40. Immunoprecipitates were eluted from the affinity resin in 2× Laemmli Buffer for 10 min at 80°C and then subjected to SDS-PAGE and Western blotting as described below.

### Immunoblotting

Extracts were run on 8–12% SDS-PAGE gels, transferred to Immobilon-P Transfer Membranes (Millipore Corp.), and visualized with antibodies specific for β-catenin (E-5) (Santa Cruz, sc-7963), β-catenin (D10A8, Cell Signaling #8480S), β-catenin (15B8, Cell Signaling, #37447), Non-Phospho (active) β-catenin (Cell Signaling, #19807S), DVL2 (Cell Signaling #3216), anti-HiBiT (Clone 30E5, Promega, CS2006A01), FLAG M2 (Sigma, F3165), anti-FLAG (Cell Signaling, D6W5B), V5 Tag (Thermo Fisher, R960-25), V5-tag (D3H8Q, Cell Signaling, #14793), GAPDH Ab (6C5) (Abcam, ab8245), NanoGlo HiBiT Blotting System (Promega, PRN2410), and UL44 (Virusys Corporation, P1202-2).

### Limiting dilution reactivation assay

CD34^+^ HPCs were infected at an MOI of 2 for 48 h. Following infection, pure populations of viable, infected (GFP-positive) CD34^+^ HPCs were isolated by Wolf Cell Sorter (Nanocellect Biomedical, Inc.) using 7AAD Viability Staining Solution (Invitrogen eBioscience) and PE-conjugated antibody specific to human CD34 (clone 581, Biolegend). Long-term cultures were maintained in Myelocult supplemented with hydrocortisone in transwells above an irradiated M2-10B4 and S1/S1 stromal cell monolayer for 12 days (53). After 12 days in long-term bone marrow culture, infected hematopoietic cell populations were serially diluted twofold in reactivation medium: RPMI supplemented with 20% FBS, 1 mM sodium pyruvate, 2 mM L-glutamine, 100 U/mL penicillin, 100 µg/mL streptomomycin, 15 ng/mL G-CSF, and 15 ng/mL GM-CSF (all cytokines purchased from R&D Systems, Minneapolis, MN, USA). Fibroblasts were examined microscopically for GFP expression up to 3 weeks. To differentiate between virus made as a result of reactivation and pre-existing virus in the cell cultures, an equal number of cells was serially diluted and plated on fibroblasts after being mechanically disrupted. The frequency of infectious center production during the culture period was measured using an extreme limiting dilution assay as previously described (53).

### Engraftment and infection of humanized mice

All animal studies were carried out in strict accordance with the recommendations of the American Association for Accreditation of Laboratory Animal Care. The protocol was approved by the Institutional Animal Care and Use Committee (protocol 0922) at Oregon Health and Science University. NOD-*scid* IL2Rγ_c_^null^ mice were maintained at a pathogen-free facility at Oregon Health and Science University in accordance with procedures approved by the Institutional Animal Care and Use Committee. Both sexes of animals were used. Humanized mice were generated as previously described (20). The animals (12–14 weeks post-engraftment) were treated with 1 mL of 4% Thioglycollate (Brewer’s Media, BD) by intraperitoneal (IP) injection to recruit monocyte/macrophages. At 24 h post-treatment, mice were infected with HCMV TB40/E-infected fibroblasts (approximately 10^5^ PFU of cell-associated virus per mouse) via IP injection. A control group of engrafted mice was mock infected using uninfected fibroblasts. The virus was reactivated as previously described (20).

### Quantitative PCR for viral genomes

Total DNA from mouse tissues was extracted using the DNAzol Reagent (Thermo Fisher Scientific) according to the manufacturer’s directions. Total DNA from primary CD34^+^ HPC cultures was extracted using TRIzol Reagent (Invitrogen) according to the manufacturer’s directions. Primers and a probe recognizing HCMV UL141 were used to quantify HCMV genomes (probe = CGAGGGAGAGCAAGTT; forward primer = 5′ GATGTGGGCCGAGAATTATGA and reverse primer = 5′ ATGGGCCAGGAGTGTGTCA). Viral genomes in humanized mice were normalized to 1-µg input DNA. Viral genomes synthesized during infection in CD34^+^ HPCs were normalized to total cell number determined using human-globin as a reference (probe = GGACAGATCCCCAAAGGACT; forward primer = 5′ TTAGGGTTGCCCATAACAGC and reverse primer = 5′ TTGGACCCAGAGGTTCTTTG). The reaction was initiated using TaqMan Fast Advanced Master Mix (Applied Biosystems) activated at 95°C for 10 min followed by 40 cycles (15 s at 95°C and 1 min at 60°C) using a StepOnePlus TaqMan PCR machine. Results were analyzed using ABI StepOne software.

### Statistical analysis

Data are expressed as the mean ± SEM. Statistical analysis was performed using GraphPad Prism (v9) for comparison between groups using Student’s *t*-test or one-way ANOVA with Tukey or Bonferroni post-test as indicated. A *P* value of <0.05 or lower was considered significant, and exact *P* values are indicated.

## SUPPLEMENTAL MATERIAL

Dataset S1 jvi.01241-23-s0001.xlsx Mass spectrometry file.

Supplemental figures and Tables jvi.01241-23-s0002.pdf Supporting text for Dataset 1 and Figure S1; Figures S1 to S8; Table S1.

ASM does not own the copyrights to Supplemental Material that may be linked to, or accessed through, an article. The authors have granted ASM a non-exclusive, world-wide license to publish the Supplemental Material files. Please contact the corresponding author directly for reuse.
