# Supplementary material for: HCMV UL8 interaction with β-catenin and DVL2 regulates viral reactivation in CD34+ hematopoietic progenitor cells
Source: J Virol. 2023 Sep 29;97(10):e01241-23. doi: 10.1128/jvi.01241-23 (PMC10617580; doi:10.1128/jvi.01241-23)
Supplement: Supplemental figures and Tables — Supporting text for Dataset 1 and Figure S1; Figures S1 to S8; Table S1. [file jvi.01241-23-s0002.pdf]

Supporting Information for

HCMV UL8 interaction with β-catenin and DVL2 regulates viral reactivation in CD34$^+$ Hematopoietic Progenitor Cells

Aaron Dirck, Nicole L. Diggins, Lindsey B. Crawford, Wilma D. Perez, Christopher J. Parkins, Hillary H. Struthers, Rebekah Turner, Andrew H. Pham, Jennifer Michell, Courtney R. Papen, Daniel Malouli, Meaghan H. Hancock and Patrizia Caposio

Corresponding author: Patrizia Caposio

Email: caposiop@ohsu.edu

Supplemental informations include:

Supporting text for Datasets S1 and Figure S1

Figures S1 to S8

Tables S1

Other supporting materials for this manuscript include the following:

Datasets S1

Liquid chromatography tandem-mass spectrometry. LC-MS/MS analysis was performed with a Thermo Scientific Easy1200 nLC (Thermo Scientific, Waltham, MA) coupled to a tribrid Orbitrap Eclipse (Thermo Scientific, Waltham, MA) mass spectrometer. In-line de-salting was accomplished using a reversed-phase trap column (100 μm × 20 mm) packed with Magic C_{18}AQ (5-μm 200Å resin; Michrom Bioresources, Auburn, CA) followed by peptide separations on a reversed-phase column (75 μm × 270 mm) packed with ReproSil-Pur C_{18}AQ (3-μm 120Å resin; Dr. Maisch, Baden-Würtemburg, Germany) directly mounted on the electrospray ion source. A 60-minute gradient using a two-mobile-phase system consisting of 0.1% formic acid in water (A) and 80% acetonitrile in 0.1% formic acid in water (B). The chromatographic separation was achieved over a 90 min gradient from 2 to 30% B over 87 min, 30 to 45% B for 10 min, 45 to 60% B for 3 min, 60 to 95% B for 2 min and held at 95% B for 11 min at a flow rate of 300 nL/minute. A spray voltage of 2300 V was applied to the electrospray tip in line with a FAIMS source using varied compensation voltage -40, -60, -80 while the Orbitrap Eclipse instrument was operated in the data-dependent mode, MS survey scans were in the Orbitrap (Normalized AGC target value 300%, resolution 240,000, and max injection time 50 ms) with a 1 sec cycle time and MS/MS spectra acquisition were detected in the linear ion trap (Normalized AGC target value of 50% and injection time 35 ms) using HCD activation with a normalized collision energy of 27%. Selected ions were dynamically excluded for 60 seconds after a repeat count of 1. Primary data analysis was performed using Proteome Discoverer 2.2 (Thermo Scientific, San Jose, CA). The data were searched against an Uniprot Human (UP000005640 from May 26, 2018) database that included common contaminants (cRAPome Jan 29, 2015). Searches were performed with settings for the proteolytic enzyme trypsin. Maximum missed cleavages were set to 2. The precursor ion tolerance was set to 10 ppm and the fragment ion tolerance was set to 0.6 Da. Dynamic peptide modifications included oxidation on methionine (+15.995 Da) and dynamic modifications on the protein N-terminus included acetyl (+42.011 Da), Met-loss (-131.040 Da on M) and Met-loss+Acetyl (-89.030 Da on M). The static modification carbamidomethyl on cysteine (+ 57.021 Da on C) was also included. Sequest HT was used for database searching. All search results were run through Percolator for scoring and identified peptides were filtered for 1% peptide-level false discovery rate using q value of 0.01.

Next generation sequencing. BAC DNA was prepared using the NucleoBond PC 100 kit (Macherey-Nagel) following the manufacturer's instructions. Illumina sequencing libraries

were generated as previously described (Burwitz BJ, et al. Cross-Species Rhesus Cytomegalovirus Infection of Cynomolgus Macaques. *PLoS Pathog.* 2016;**12**:e1006014. doi: 10.1371/journal.ppat.1006014). Briefly, DNA was fragmented using an S2 Sonicator and was then converted to libraries using the standard TruSeq protocol. Libraries were examined on a Bioanalyzer (Agilent) and the concentration was determined using real time PCR and SYBR Green fluorescence. NGS was performed using an iSeq NGS System (Illumina). Libraries were loaded into an iSeq reagent cartridge at a concentration of 9 pM and single read sequencing was performed for 300 cycles with 6 additional cycles of index reads. The resulting data was imported into Geneious and the sequencing reads were trimmed of all regions exceeding the error probability limit of 0.1% to minimize sequencing errors. All reads with a total length of fewer than 50 bp after quality control were eliminated from further analysis to increase the likelihood of specific alignments during *de novo* and reference guided assemblies. Viral genomes were first *de novo* assembled using the processed sequencing data, and subsequently all reads were aligned to the generated consensus sequence in a reference guided assembly to examine potential SNPs.

A

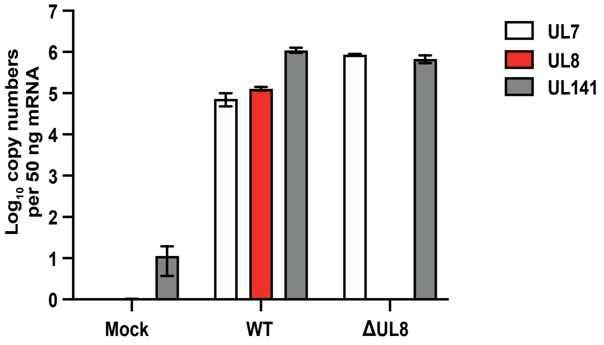

B

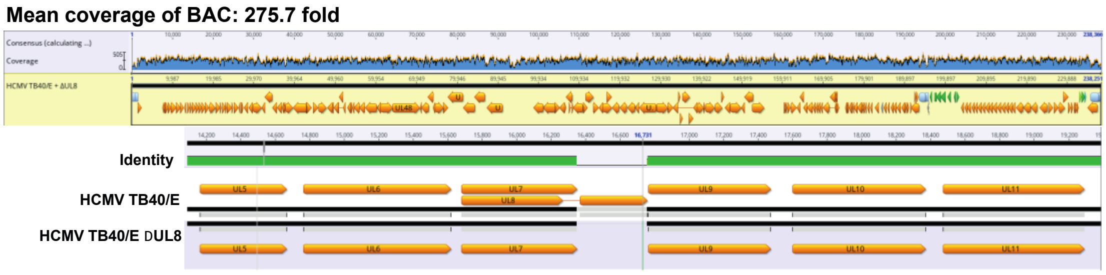

Fig. S1. Genome analysis of HCMV TB40/E ΔUL8. A) NHDF cells were mock-infected or infected with HCMV TB40/E (WT) or TB40/EΔUL8 (ΔUL8) at MOI of 2 for 72h. RNA was isolated using Trizol and qRT-PCR for *UL7, UL8, and UL141* was performed. Values are means±standard error of the means (SEM) (error bars). B) The top panel shows the sequencing coverage map for the TB40/E ΔUL8 (BAC) graphically depicted as number of reads per nucleotide position. The ORF map is shown below with the BAC cassette highlighted in green. Genome alignments of HCMV TB40/E and HCMV TB40/E ΔUL8 are shown below with the region that differs in HCMV TB40/E ΔUL8. The green bar indicates the percentage of nucleotide identity between both virus sequences with green being 100% identical.

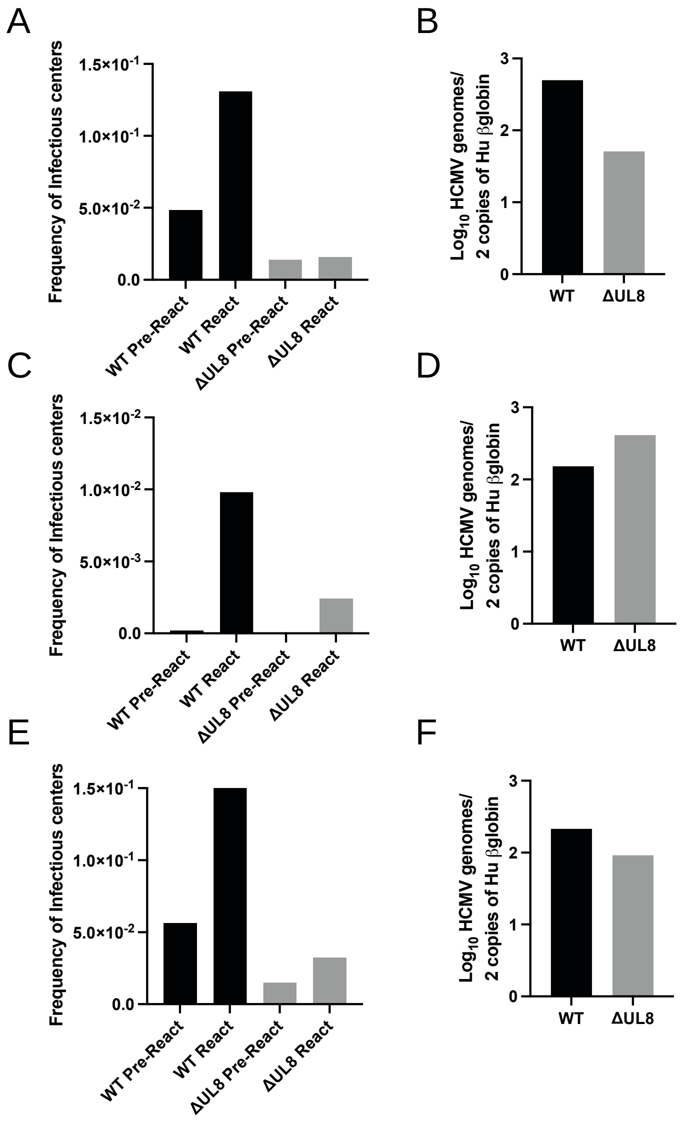

Fig. S2. UL8 is required for reactivation, but not genome maintenance. $CD34^+$ HPCs were infected with HCMV TB40/E (WT) or TB40/EΔUL8 (ΔUL8) for 48h, sorted for pure $CD34^+$ GFP^+HPCs and plated for long-term culture on stromal cell support. After 12 days (14 dpi), reactivation was assessed by co-culture on fibroblasts from three independent experiments (A, C, E). DNA from a subset of cells was prepared using the two-step TRIZOL method and viral genomes analyzed by qPCR (B, D, F).

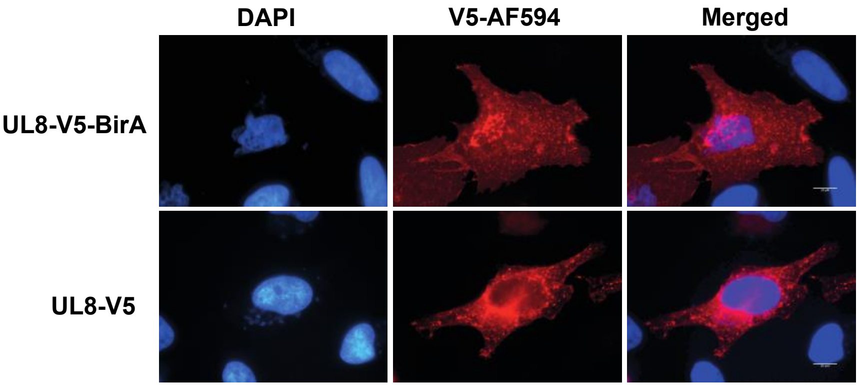

Fig. S3. TurboID does not alter UL8 cellular localization. HeLa cells were transfected with pCDNA3.1UL8-V5 or pCDNA3.1-UL8-V5-TbID for 24 hours. Cells were fixed in 4% formaldehyde and permeabilized with PBS + 0.1% Triton X-100. Cells were stained with 1:200 α-V5 (Cell Signaling #13202) and 1:400 goat anti-rabbit conjugated with AlexaFluor 594 (Thermo Fisher) and mounted with DAPI Fluoromount-G (Southern Biotech, Cat no. 0100-20). Immunofluorescent images were captured on Life Technologies EVOS FL Auto equipped with an Olympus UPlanSApo 100x/1.40na Oil Objective.

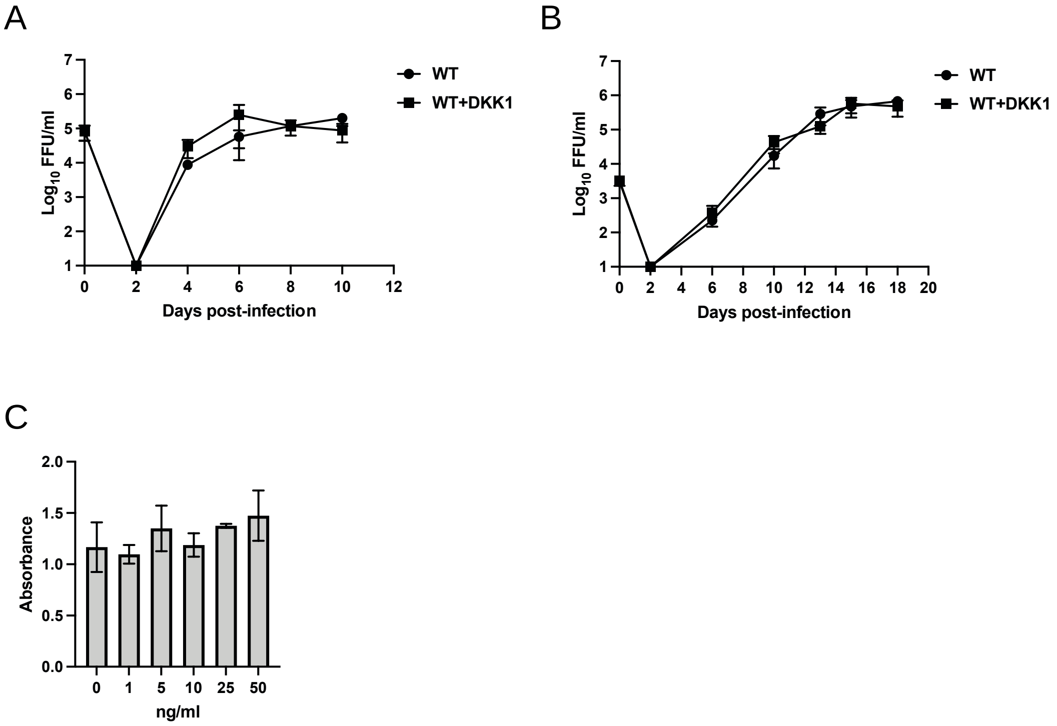

Fig. S4. Treatment with DKK1 does not alter HCMV lytic replication. A) NHDF cells were infected at an MOI of 1 and then left untreated or treated with DKK1 (100 ng/ml). Supernatants were harvested at the indicated times post-infection and titrated by TCID50. B) NHDF cells were infected at an MOI of 0.01 and then left untreated or treated with DKK1 (100 ng/ml). Supernatants were harvested at the indicated times post-infection and titrated by TCID50. C) $CD34^+$ HPCs were incubated with increasing concentrations of DKK1 for 7 days. Colorimetric assay (WST-1 based, Roche) was used to perform the cytotoxicity assay according to the manufacturer’s directions. Values are means±standard error (SD) (error bars).

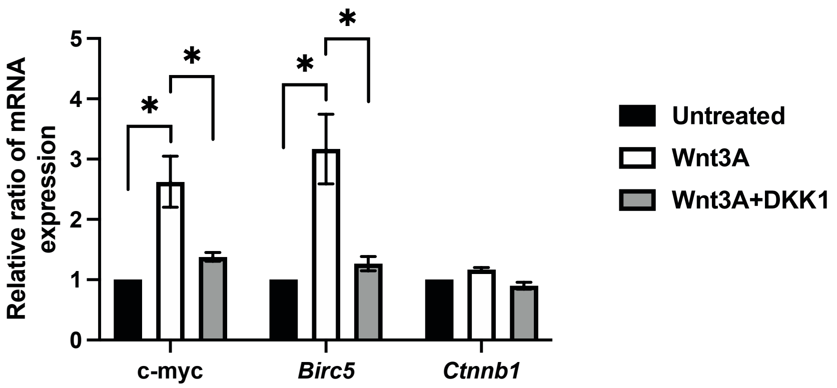

Fig. S5 $CD34^+$ HPCs were treated with Wnt 3A (100 ng/ml) in the presence or absence of DKK1 (50 ng/ml) for 24h. RNA was isolated using Trizol and qRT-PCR for *c-myc*, *Birc5*, and *Ctnnb1* was performed. Values are means±standard error of the means (SEM) (error bars) compared to untreated for three independent experiments. Statistical significance was determined using one way-ANOVA with Tukey's multiple comparison test (*, $p<0.05$).

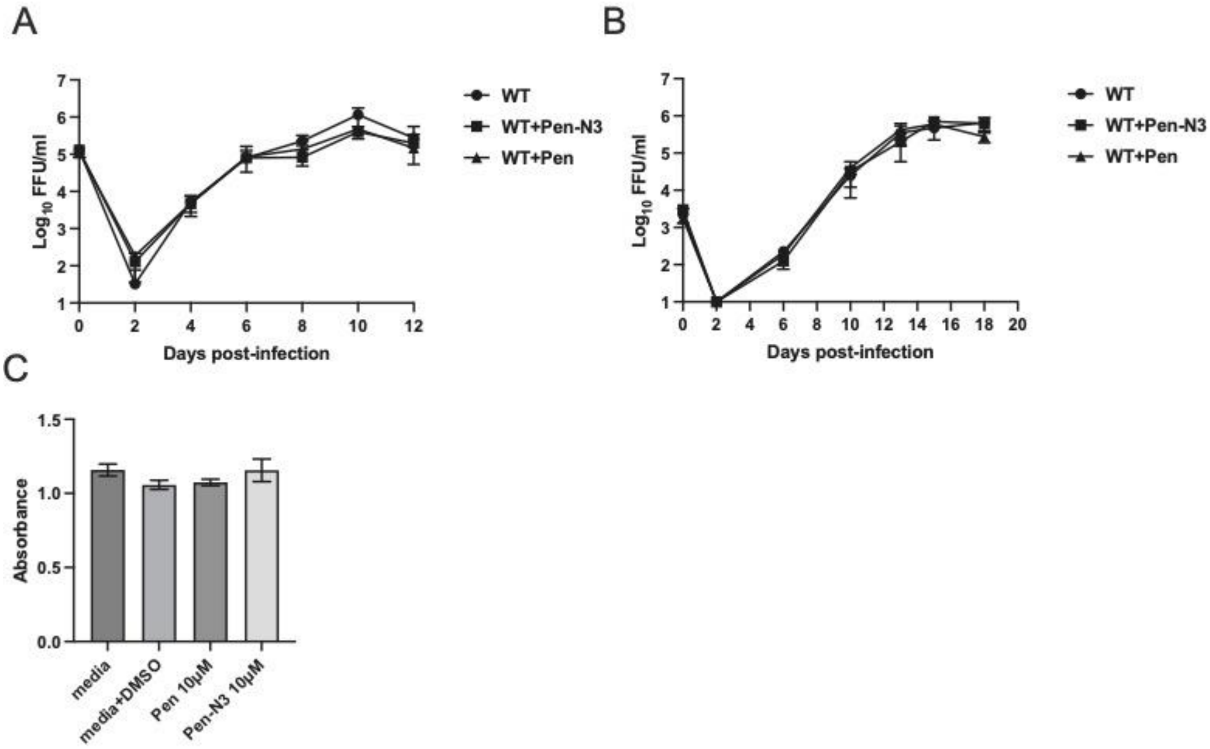

Fig. S6. Pen-N3 peptide does not alter HCMV lytic replication and is not cytotoxic. A) NHDF cells were infected with HCMV WT at an MOI of 1, and then treated with Pen-N3, Pen (10 μM) or DMSO. Supernatants were harvested at the indicated times post-infection and titrated by TCID50. B) NHDF cells were infected with HCMV WT at an MOI of 0.01, and then treated with Pen-N3, Pen (10 μM) or DMSO. Supernatants were harvested at the indicated times post-infection and titrated by TCID50. C) $CD34^+$ HPCs were incubated in media, media + DMSO, Pen (10 μM) or Pen-N3 (10 μM) for 7 days. Colorimetric assay (WST-1 based, Roche) was used to perform the cytotoxicity assay according to the manufacturer’s directions. Values are means±standard error (SD) (error bars).

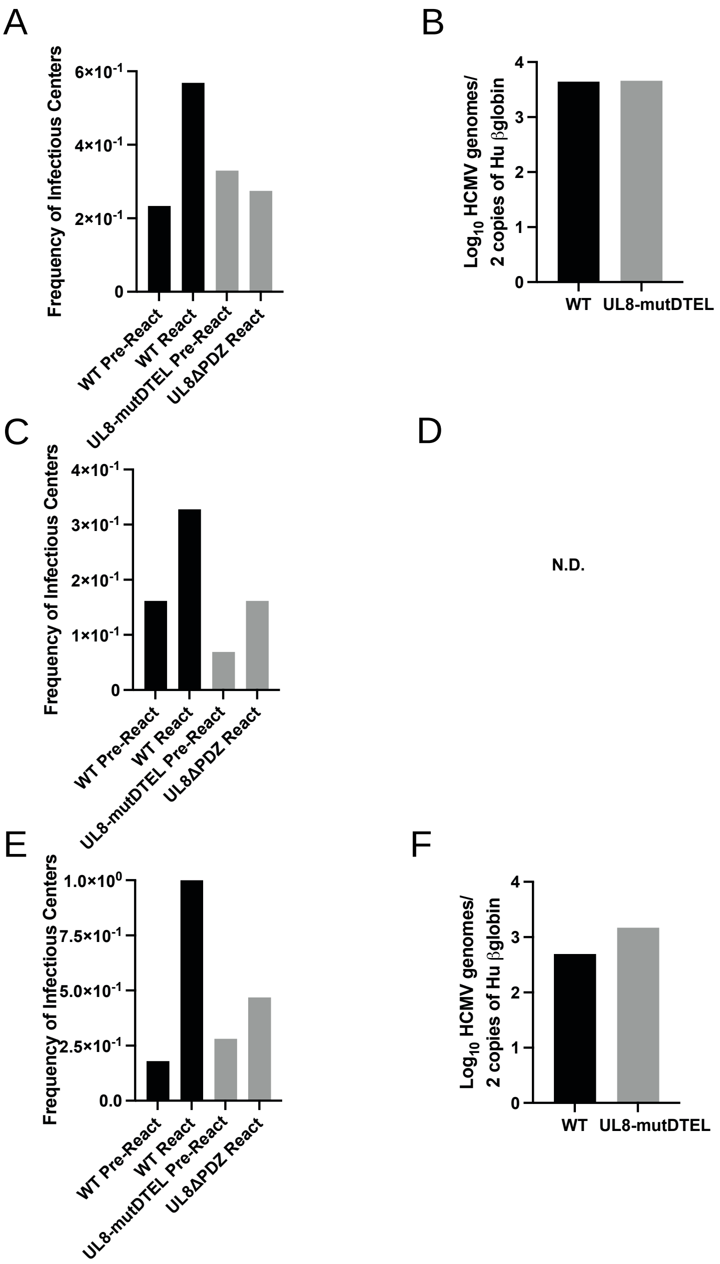

Fig. S7. CD34^{+} HPCs were infected with HCMV TB40/E UL8-V5 (WT) or TB40/E UL8-mutDTEL-V5 (UL8-mutDTEL) for 48h, sorted for pure CD34^{+} GFP^{+}HPCs and plated for long-term culture on stromal cell support. After 12 days (14 dpi), reactivation was assessed by co-culture on fibroblasts from three independent experiments (A, C, E). DNA from a subset of cells was prepared using the two-step TRIZOL method and viral genomes analyzed by qPCR (B, D, E).

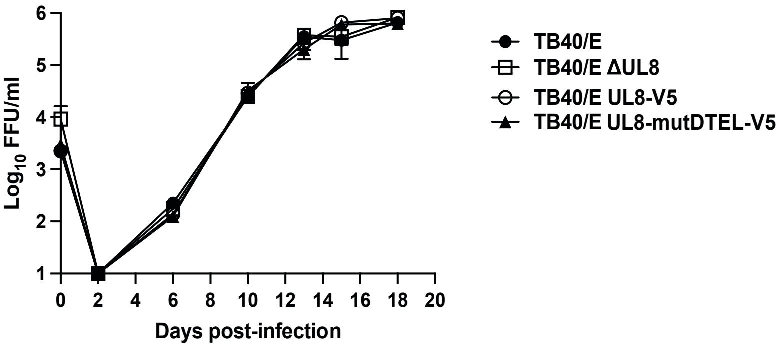

Fig. S8. Neither deletion of UL8 nor mutation of the PDZ domain impact HCMV lytic replication. NHDF cells were infected at an MOI of 0.01 with TB40/E, TB40/EΔUL8, TB40/E UL8-V5, TB40/E-mutDTEL-V5. Supernatants were harvested at the indicated times post-infection and titrated by TCID50.

Table S1. Oligonucleotides used for BAC mutagenesis

Primer designation	Sequence (5' to 3')
Galk/kan UL7 for	GGCACTGTTTGAGCATGACTGTTTCCAAACCGTAACGTGGTAAATAA ATCCCTGTTGACAATTAATCATCGGCATAG
Galk/kan UL8 rev	TTCCGACACAGATAGCCCCGATGTAGTCATTGTTGGTACAAAACCTT CTCCTCAGCAAAAGTTCGATTTA*
HiBiT-UL8 (DOG) for	GGCACTGTTTGAGCATGACTGTTTCCAAACCGTAACGTGGTAAATAA ATCATGGCTTCCGACGTGGTTC
HiBiT-UL8 (DOG) rev	TTCCGACACAGATAGCCCCGATGTAGTCATTGTTGGTACAAAACCTT CTCCCAAGG CCATGTGCGTGTTGTT[#]
GalK/kan UL8 for	GGAAAGACAGCAGAGAGTACGGACAAGTGTTTATGACGGACACAGA ACTGCTGTTGACAATTAATCATCGGCATAG
Galk/kan UL8 rev	ATTGTCATCCATAGAAATAACCGCAAACACTTCTTAGACATCATAGTA TACTCAGCAAAAGTTCGATTTATTCAAC
UL8-V5-TbID (DOG) for	GGAAAGACAGCAGAGTACGGACAAGTGTTTATGACGGACACAGAAC TGGGCAAGCCCATCCCCAACCC
UL8-V5-TbID (DOG) rev	ATTGTCATCCATAGAAATAACCGCAAACACTTCTTAGACATCATAGTA TATTACTTTTCGGCAGACCGCAGACTGA
UL8-mutDTEL-V5-TbID oligo	GGAAAGACAGCAGAGAGTACGGACAAGTGTTTATGACGGCCGCAGC AGCGGGCAAGCCCATCCCCAACCCCTGCT
Galk/kan UL22A for	AGATGTCGTCACCCAAGGTATTTAACGGCACACAGCCAGACGCGTTC GTCAGCAGCGACGCCGACAAGACCTCAGCCCTGTTGACAATTAATCA TCGGCA
Galk/kan UL22A rev	TTAGAGCAAAACCTTACAGCTTTTTAATAAAAAACAAGGTAGTCAACA TAATCGTTAACCCTTGGGGTCTGCTGCTCAGCAAAAGTTCGATTTA
HiBiT-UL22A (DOG) for	AGATGTCGTCACCCAAGGTATTTAACGGCACACAGCCAGACGCGTTC GTCAGCAGCGACGCCGACAAGACCTCAGCATGGCTCGGAGGCTATG GATCTTG
HiBiT-UL22A (DOG) rev	TTAGAGCAAAACCTTACAGCTTTTTAATAAAAAACAAGGTAGTCAACA TAATCGTTAACCCTTGGGGTCTGCTG TTACTTGTCGTCATCGTCTTT GTAGT
HCMVTB40/EΔUL8S	CACGCACATGGCCTTGGTAGGTGTTGTCGTGTTTCTAGCCCTAATAG TTGTTTGTATTATGGGGTGGTGGAAGTTGTTGTGTAGTAAACCAAAGT TATAGTAGGGATAACAGGGTAATAAG
HCMVTB40/EΔUL8AS	ATTGTCATCCATAGAAATAACCGCAAACACTTCTTAGACATCATAGTA TACTATAACTTTGGTTTACTACACAACAACTTCCACCACCCCATAATA CAAAAGAGCGCTTTTGAAGCTGG

*this primer binds at the end of UL8 spliced sequence; [#] this primer binds upstream of UL8 spliced sequence.
